# Compact ultrabroadband light-emitting diodes based on lanthanide-doped lead-free double perovskites

**DOI:** 10.1038/s41377-022-00739-2

**Published:** 2022-03-08

**Authors:** Shilin Jin, Renfu Li, Hai Huang, Naizhong Jiang, Jidong Lin, Shaoxiong Wang, Yuanhui Zheng, Xueyuan Chen, Daqin Chen

**Affiliations:** 1 College of Physics and Energy, Fujian Normal University, Fujian Provincial Key Laboratory of Quantum Manipulation and New Energy Materials, Fuzhou, 350117 China; 2 Fujian Science & Technology Innovation Laboratory for Optoelectronic Information, Fuzhou, 350116 China; 3 CAS Key Laboratory of Design and Assembly of Functional Nanostructures, Fujian Key Laboratory of Nanomaterials and State Key Laboratory of Structural Chemistry, Fujian Institute of Research on the Structure of Matter, Chinese Academy of Sciences, Fuzhou, Fujian 350002 China; 4 Fujian Provincial Collaborative Innovation Center for Advanced High-Field Superconducting Materials and Engineering, Fuzhou, 350117 China; 5 Fujian Provincial Engineering Technology Research Center of Solar Energy Conversion and Energy Storage, Fuzhou, 350117 China; 6 College of Chemistry, Fuzhou University, Fuzhou, 350116 China

**Keywords:** Inorganic LEDs, Optical materials and structures

## Abstract

Impurity doping is an effective approach to tuning the optoelectronic performance of host materials by imparting extrinsic electronic channels. Herein, a family of lanthanide (Ln^3+^) ions was successfully incorporated into a Bi:Cs_2_AgInCl_6_ lead-free double-perovskite (DP) semiconductor, expanding the spectral range from visible (Vis) to near-infrared (NIR) and improving the photoluminescence quantum yield (PLQY). After multidoping with Nd, Yb, Er and Tm, Bi/Ln:Cs_2_AgInCl_6_ yielded an ultrabroadband continuous emission spectrum with a full width at half-maximum of ~365 nm originating from intrinsic self-trapped exciton recombination and abundant 4f–4f transitions of the Ln^3+^ dopants. Steady-state and transient-state spectra were used to ascertain the energy transfer and emissive processes. To avoid adverse energy interactions between the various Ln^3+^ ions in a single DP host, a heterogeneous architecture was designed to spatially confine different Ln^3+^ dopants via a “DP-in-glass composite” (DiG) structure. This bottom-up strategy endowed the prepared Ln^3+^-doped DIG with a high PLQY of 40% (nearly three times as high as that of the multidoped DP) and superior long-term stability. Finally, a compact Vis–NIR ultrabroadband (400~2000 nm) light source was easily fabricated by coupling the DiG with a commercial UV LED chip, and this light source has promising applications in nondestructive spectroscopic analyses and multifunctional lighting.

## Abstract (web summary)

Ln:Cs_2_Ag(In/Bi)Cl_6_@glass composite yielding a Vis–NIR continuous spectrum is used to construct an ultrabroadband lighting source.

## Graphical abstract

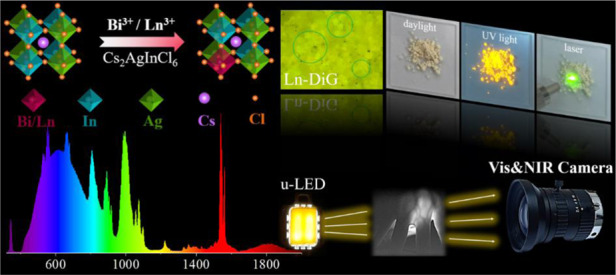

## Introduction

Ultrabroadband light sources that emit over an extremely wide spectral range are of great interest in many fields, such as photonics, medical treatment, high-capacity optical data communications, ultraprecision metrology, and spectroscopy^1–7^. Conventionally, halogen tungsten lamps (HTLs) are used in most studies and applications, but they generate large amounts of heat and have limited operational lifetimes^8^. Recently, superluminescent diodes (SLDs)^9^, ultrabroadband semiconductor lasers (UBSLs)^10^, laser-driven light sources (LDLSs)^11^, supercontinuum light sources (SCLSs)^12^, and other sources of light have been developed to satisfy the needs of various applications. SLDs are fabricated by elaborately coupling many light-emitting diodes (LEDs) with different emission wavelengths. UBSLs adopt a quantum cascade configuration, where several dissimilar inter-subband (IS) optical transitions cooperate to provide broadband optical gain. LDLSs use high-power lasers to energize high-intensity xenon (Xe) plasma, which produces broadband radiation. SCLSs are generated in a photonic crystal fiber pumped by a femtosecond laser. Notably, each of these approaches has intrinsic drawbacks, such as complex structure design for coupling multiple LEDs, difficulty in tailoring IS transitions, low efficiency, and expensive laser triggers.

Lanthanide (Ln^3+^) or rare-earth ion incorporation or doping is considered a promising approach to imparting and tailoring the optical and optoelectronic performances of inorganic materials spanning the ultraviolet (UV), visible (Vis), near-infrared (NIR), and middle-infrared (MIR) regions, improving their abundant intermediate excited states^13^. For example, Er-doped fiber amplification (EDFA), praseodymium-doped fiber amplifier (PDFA), and thulium-doped fiber amplifier (TDFA) with broadband NIR emissions have been successfully applied in optical telecommunication^14–16^. However, the parity-forbidden 4f–4f transition of Ln^3+^ leads to weak light absorption, which limits the practical application of these materials. Sensitizing Ln^3+^ emission via semiconductor quantum dots (QDs) can effectively address this difficult challenge because QDs undergo efficient allowed band-to-band absorption and have tuneable bandgaps^17^. Recently, various lanthanide ions have been successfully doped into the hottest lead-halide perovskite QD (PeQD) lattices to endow them with optical functionality^18–21^. Unfortunately, their use of toxic lead and their poor stability to heat, water, electric fields, and light hamper their commercialization and industrialization on a large scale^22,23^. As an alternative, lead-free halide double perovskites (DP_S_) have drawn extensive attention for their fascinating optical performance and excellent stability^24–26^. Among the DP family, Cs_2_AgInCl_6_ has been in the spotlight for its direct bandgap characteristics and various alloy compositions with mono- and trivalent cations other than Ag^+^ and In^3+ ^^27–29^. For example, Cs_2_AgInCl_6_ alloyed with 40% Na^+^ and 0.04% Bi^3+^ doping emits warm white light with ~86% photoluminescence quantum yield (PLQY) and works beyond 1000 h without obvious PL loss^30^. The underlying mechanism of the broadband luminescence in the visible (400~800 nm) spectral range proceeded via the recombination of self-trapped excitons (STEs), which mainly arose from the strong Jahn–Teller distortion of the AgCl_6_ octahedron triggered by Na^+^ dopants^30^. Since then, several successful examples of DPs doped with Ln^3+^ ions (Table S1), including La-, Tb-, Er-, and Yb-doped Cs_2_AgInCl_6_, have been reported, primarily for their potential applications in white-light-emitting diodes (wLEDs), NIR-LEDs with specific wavelengths, scintillators, anticounterfeiting techniques, and X-ray detection and imaging^31–45^.

Inspired by the studies described above, we intend to explore the possibility of combining the STE recombination of Cs_2_AgInCl_6_ with Ln^3+^ dopant-induced extra light-emitting channels to produce lead-free Vis–NIR (400~2000 nm) ultrabroadband emissive DPs for the first time. To our knowledge, there has been no report on Ln^3+^-doped DPs aimed at the application of ultrabroadband light sources. Herein, a family of Ln^3+^ dopants (Ln=La–Lu) with Bi^3+^ ions is successfully incorporated into Cs_2_AgInCl_6_ DPs. The Bi/Ln (Ln=Nd, Yb, Er, and Tm):Cs_2_AgInCl_6_ samples can yield both visible STE radiation and multiple NIR Ln^3+^ 4f–4f emissions under UV light excitation, and the related energy transfer mechanisms are systematically discussed. We further provide a strategy of dispersing Nd:Cs_2_AgInCl_6_, Yb/Er:Cs_2_AgInCl_6_, and Yb/Tm:Cs_2_AgInCl_6_ DPs into an inorganic glass matrix to prevent the depletion of nonradiative migration from the co-doping of multiple Ln^3+^ ions in a sole DP host, leading to detrimental luminescent quenching. To the best of our knowledge, this is the first report on a DP-in-glass (DiP) composite. The DiP composite with superior stability and a high PLQY of ~40% can be coupled with a commercial UV LED chip to construct an ultrabroadband LED (u-LED) device. Finally, we demonstrate its promising applications in spectroscopic analysis and multifunctional lighting. Notably, compared with previously reported ultrabroadband light sources, this u-LED device shows the advantages of cost-effective fabrication, compactness, excellent optical performance and extremely long-term stability.

## Results and discussion

Bi/Ln-doped Cs_2_AgInCl_6_ DPs were synthesized via a modified coprecipitation method (Fig. S1, Experimental Section) previously reported by Volonakis et al.^27^. In a typical synthesis, stoichiometric amounts of InCl_3_, AgCl, BiCl_3_, and LnCl_3_ are dissolved in hydrochloric acid at 100 °C. Upon the addition of CsCl solution, a precipitate forms after 20 min of reaction. Notably, a shaded glass bottle is needed in the synthesis of DPs to prevent the silver chloride from being oxidized (Supporting Information, Movie 1). We first prepared Cs_2_AgInCl_6_ DPs doped with only Bi^3+^ and investigated the influence of Bi^3+^ doping content on the structure and optical properties. The X-ray diffraction (XRD) patterns showed that the pure cubic phase was retained after Bi^3+^ doping (Fig. S2a). With increasing Bi^3+^ content, the diffraction peaks shifted slightly to a lower angle (Fig. S2b), indicating the substitution of In^3+^, which has a small ionic radius (*r* = 0.80 Å, CN = 6), with Bi^3+^, which has a large ionic radius (*r* = 1.02 Å, CN = 6). PL originating from STE recombination for the Cs_2_AgInCl_6_ DPs is quite weak, and the corresponding PLQY is lower than 1%; Bi^3+^ doping will significantly improve the PL intensity, decay lifetime, and PLQY up to ~25% for STE emission (Fig. S2c, Fig. S2d, Fig. S3). The optimal Bi^3+^ nominal doping content is 0.048 mmol (Fig. S3), which corresponds to the actual content of ~15 mol% determined by inductively coupled plasma–mass spectrometry (ICP–MS) (Table S2). The excitation power (intensity)- and temperature-dependent PLQYs of Bi:Cs_2_AgInCl_6_ were determined. The PLQY remained unchanged with increasing excitation power and was insensitive to excitation intensity (Fig. S3d). However, with increasing temperature, the PLQY showed a tendency to decrease owing to thermal quenching of STE recombination (Fig. S3e). The normalized PL/PLE spectra show redshifting of both the emission and excitation bands with increasing Bi^3+^ content (Fig. S2e, S2f), indicating the Bi^3+^-doping-induced modification of the band-edge structure of Cs_2_AgInCl_6_ DPs^46–49^. We prepared additional Bi^3+^/Er^3+^ codoped samples with various Bi^3+^ contents and fixed Er^3+^ contents. All the samples were assigned to the cubic Cs_2_AgInCl_6_ phase and exhibited sharp emission peaks corresponding to the Er^3+^: ^2^H_11/2_ → ^4^I_15/2_, ^4^S_3/2_ → ^4^I_15/2_, ^4^F_9/2_ → ^4^I_15/2_, and ^4^I_9/2_ → ^4^I_15/2_ transitions in the visible spectral region upon excitation with 250~450 nm light (Fig. S4), confirming the incorporation of the Er^3+^ dopants into the DP host. With the addition of Er^3+^ ions, the total emitting intensity was greater than that of the sample doped with only Bi^3+^ owing to the superposition of the additional Er^3+^ emissions, and the optimal Bi^3+^ content remained at 0.048 mmol (Fig. S5). As evidenced in Fig. S6, changing the Er^3+^ content does not greatly enhance the Er^3+^ emission intensity. Compared to that of the sample doped with only Bi, the PLQY for the Er/Bi codoped sample is greater; however, the PLQY does not noticeably differ for different Er^3+^-doped samples (Fig. S6). These results indicate the doping saturation of Er^3+^ ions, which makes high-content Er^3+^ doping difficult. Indeed, the ICP–MS (Table S2) data verify the saturated doping of Er^3+^ in DPs with the highest content of ~2.5 mol%, even when the nominal Er/In doping ratio reaches as high as 3. Similarly, for the Yb/Er co-doped sample, the total doping content of both Yb and Er is also close to 2.5 mol% (Table S2). Indeed, intense upconversion luminescence can be detected from the Bi/Yb/Ln (Ln=Er, Ho, or Tm):Cs_2_AgInCl_6_ samples under 980 nm laser excitation (Fig. S7), further confirming the incorporation of Ln^3+^ ions in the DP host. Notably, it is easy to incorporate many Bi^3+^ ions into Cs_2_AgInCl_6_ because the structure of Cs_2_Ag(In/Bi)Cl_6_ is a stable solid solution^50^, while doping Ln^3+^ ions into Cs_2_AgInCl_6_ is difficult because the electronic configurations of the Ln^3+^ ions and In^3+^ ions are quite different.

**Fig. 1:**
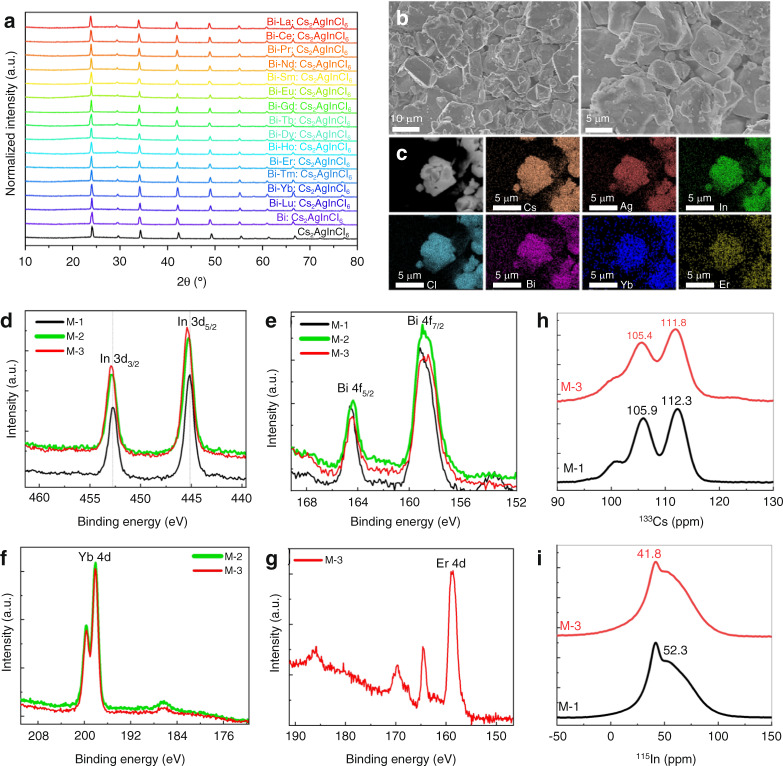
Structural characterization of Bi/Ln:Cs_2_AgInCl_6_ DPs. **a** XRD patterns of Bi/Ln:Cs_2_AgInCl_6_ DPs. **b** SEM images of a typical Bi/Yb/Er:Cs_2_AgInCl_6_ sample and (**c**) the corresponding EDX mappings. **d**–**g** XPS spectra of the In 3d, Bi 4f, Yb 4d, and Er 4d orbitals of M-1 (Bi:Cs_2_AgInCl_6_, black line), M-2 (Bi/Yb:Cs_2_AgInCl_6_, green line), and M-3 (Bi/Yb/Er:Cs_2_AgInCl_6_, red line) samples. **h** ^133^Cs and **i** ^115^In NMR spectra of the M-1 and M-3 samples

The XRD patterns for the whole family of Bi–Ln (Ln=La–Lu)-doped DP samples are provided in Fig. 1a. Introducing Ln^3+^ dopants does not induce the formation of impurity phases for all the products, and the diffraction peaks were assigned to the cubic Cs_2_AgInCl_6_ phase. The diffraction peak slightly shifted to a smaller angle after the substitution of In^3+^ by Ln^3^; this shift was due to the gradual increase in ionic radius from Lu^3+^ to La^3+^ (Fig. S8), suggesting successful incorporation of Ln^3+^ dopants into the Cs_2_AgInCl_6_ crystalline lattice. Taking Yb/Er:Cs_2_AgInCl_6_ as a typical example, the scanning electron microscopy (SEM) images show that the products are micron-sized crystals with polygonal shapes (Fig. 1b). Energy-dispersive X-ray (EDX) mappings confirm the uniform distribution of all the elements of Cs, Ag, In, Cl, Bi, Yb, and Er (Fig. 1c). The actual elemental content was consistent with the experimental scheme (Fig. S6d, Table S3). X-ray photoelectron spectroscopy (XPS) measurements on the M-1(Bi:Cs_2_AgInCl_6_), M-2 (Bi/Yb:Cs_2_AgInCl_6_), and M-3 (Bi/Yb/Er Cs_2_AgInCl_6_) samples were performed (Fig. S9). Typical Cs 3d, Ag 3d, In 3d, and Cl 2p signal peaks (Fig. S9, Fig. 1d) were detected in the high-resolution XPS spectra, and extra Bi 4f (Fig. 1e), Yb 4d (Fig. 1f), and Er 4d (Fig. 1g) peaks were observed for the Bi/Yb/Er:Cs_2_AgInCl_6_ sample. Additionally, as shown in Fig. 1d, the shifting of the In 3d peak toward higher energy is observed after Bi/Yb/Er doping. Generally, the substitution of In^3+^ ions by Bi^3+^ and Ln^3+^ dopants in Cs_2_AgInCl_6_ leads to lattice expansion and thus a slight shift to a higher binding energy of the In^3+^ 3d XPS peak. Similar results have been observed in Bi/Tb-doped Cs_2_AgInCl_6_ samples and have been previously reported.^38^ Furthermore, magic-angle-spinning (MAS) solid-state nuclear magnetic resonance (NMR) measurements for ^133^Cs and ^115^In were performed on the Bi:Cs_2_AgInCl_6_ and Bi/Yb/Er:Cs_2_AgInCl_6_ samples. The doping of paramagnetic Yb^3+^ and Er^3+^ induced a shift of the ^133^Cs doublet peaks from 105.9/112.3 ppm to 105.4/111.8 ppm (Fig. 1h). This shift is ascribed to the delocalization of the extra electron density of Yb^3+^ and Er^3+^ (spin I = 1/2 and 3/2) on the Cs^+^ ion (i.e., the Fermi-contact interaction)^41,51–53^. The two samples show the peaks of ^115^In at 41.8 ppm and 52.3 ppm (Fig. 1i), and the relative peak intensity decreases at 41.8 ppm after Ln doping^53^. The results of the above two changes further verify the successful incorporation of rare-earth ions into the Cs_2_AgInCl_6_ DP lattice.

**Fig. 2:**
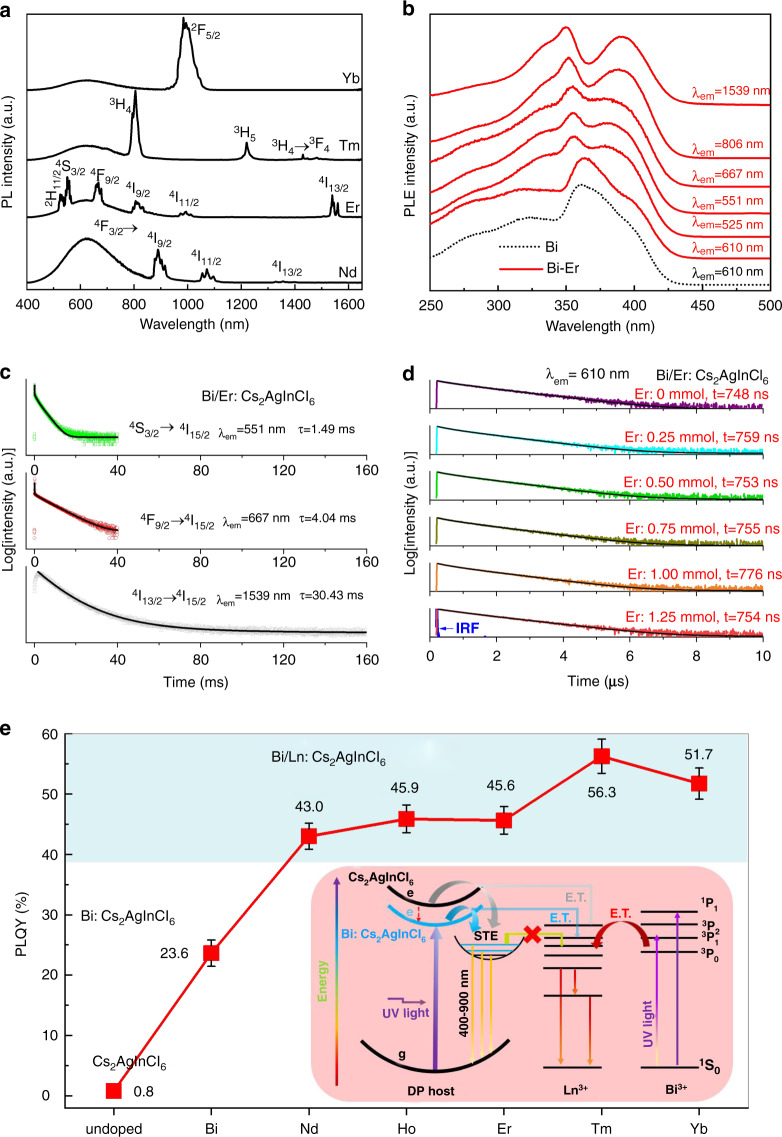
Spectroscopic characterization and energy-transfer processes in Bi/Ln:Cs_2_AgInCl_6_ DPs. **a** PL spectra of the Bi/Ln (Ln=Nb, Er, Tm, and Yb): Cs_2_AgInCl_6_ samples. **b** PLE spectra of the Bi:Cs_2_AgInCl_6_ and Bi/Er:Cs_2_AgInCl_6_ samples obtained by monitoring different emitting wavelengths. PL decay curves obtained by monitoring (**c**) Er^3+^ emissions and (**d**) STE recombination in the Bi/Er:Cs_2_AgInCl_6_ samples. IRF represents the instrumental response function. **e** PLQYs for a series of the Bi/Ln:DPs. The inset shows the proposed energy-transfer mechanisms and emission processes involving both STE recombination and Ln^3+^ 4f–4f transitions

We recorded the PL spectra of all the Bi/Ln codoped Cs_2_AgInCl_6_ samples. Under 350 nm UV light excitation, typical narrow emission peaks assigned to Ln^3+^ 4 f → 4 f transitions appeared in addition to the broadband emission of STE for the Pr^3+^, Nd^3+^, Sm^3+^, Tb^3+^, Dy^3+^, Ho^3+^, Er^3+^, Tm^3+^, and Yb^3+^-doped samples (Fig. S10). All the samples exhibited bright luminescence, and the slight difference in emission color is due to the combination of STE emissive color and Ln^3+^ color (Fig. S11). The corresponding Ln^3+^ 4f → 4f electronic transitions and emitting wavelengths are tabulated in Table S4. For the other Ln^3+^ (Ln=La, Ce, Eu, Gd, and Lu)-doped samples, only STE emission was observed. La^3+^, Ce^3+^, Gd^3+^, and Lu^3+^ dopants with outer-shell electronic configurations of 4f^0^, 4f^1^, 4f^7^, and 4f^14^ do not have suitable energy levels falling inside the bandgap of DP^13^, and no energy transfer from DP to them occurs. Eu^3+^ dopants easily produce electron trapping states in empty f-orbitals in the bandgap^36,39^, which act as quenching centers. Importantly, as demonstrated in Fig. 2a, the combination of STE broadband visible emission (400–800 nm) with a series of narrowband NIR emissions from Ln^3+^ ions (Yb^3+^: ^2^F_5/2_ → ^2^F_7/2_; Tm^3+^: ^3^H_4_ → ^3^H_6_, ^3^H_5_ → ^3^H_6_, ^3^H_4_ → ^3^F_4_; Er^3+^: ^4^I_11/2_ → ^4^I_15/2_, ^4^I_13/2_ → ^4^I_15/2_; Nd^3+^: ^4^F_3/2_ → ^4^I_9/2_, ^4^F_3/2_ → ^4^I_11/2_, ^4^F_3/2_ → ^4^I_13/2_) is feasible to realize an ultrabroadband continuous emission spectrum. Taking Bi/Er:Cs_2_AgInCl_6_ DP as a typical example, the PL excitation (PLE) spectra were recorded by monitoring the various emissions of Er^3+^ (Fig. 2b). The PLE spectra corresponding to the broad visible-light emission of STE at 610 nm are similar for both the Bi:Cs_2_AgInCl_6_ and Bi/Er:Cs_2_AgInCl_6_ samples, but are quite different from those of the Er^3+^ emissions at 525 nm, 551 nm, 667 nm, 806 nm, and 1539 nm, respectively. In particular, the PLE spectrum for 1539 nm NIR emission without the interference of STE luminescence shows two typical absorption bands rather than semiconductor-like band-to-band absorption (Fig. 2b). These two absorption bands can be assigned to Bi^3+^: 6s^2^ → 6s6p (^1^S_0_ → ^3^P_1_, ^1^S_0_ → ^1^P_1_) absorption transitions^54^. In fact, similar phenomena can be found in the PLE spectra of the other Ln^3+^ (Nd, Ho, Tm, and Yb) NIR emissions (Fig. S12). These results indicate that the excitation of Ln^3+^ ions in Cs_2_AgInCl_6_ DP is mainly achieved via energy transfer from Bi^3+^ ions rather than the STE state (DP host). To verify this, we detected the PL decay curves for the STE emission of the Bi-doped and Bi/Er codoped samples. The decay curves of the Er^3+^ emissions (Fig. 2c) exhibited typical parity-forbidden 4f–4f transitions with a long lifetime on a millisecond scale (1.49 ms for Er^3+^: ^4^S_3/2_ → ^4^I_15/2_, 4.04 ms for Er^3+^: ^4^F_9/2_ → ^4^I_15/2_, and 30.43 ms for Er^3+^: ^4^I_13/2_ → ^4^I_15/2_), while the decay lifetime for STE emission was only ~750 ns owing to the strong Jahn–Teller distortion-induced electron–hole recombination (Fig. 2d). Notably, the decay lifetime for STE recombination shows no obvious change with increasing Er^3+^ doping content, indicating that there was no direct energy transfer from STE to the Ln dopants.

Herein, we propose a possible mechanism that describes the Ln^3+^ emissions in Bi/Ln:Cs_2_AgInCl_6_ DP, as demonstrated in Fig. 2e. Cs_2_AgInCl_6_ DP is a direct bandgap semiconductor that undergoes a parity-allowed higher-order transition between the third valence band (VB) level and conductive-band minima (CBM) at ~4.8 eV^27,28^. Interestingly, Bi^3+^ doping decreases the excitation energy to the UV region (extra-absorption energy state at ~377 nm, Fig. S3c) but no other PL peak, except STE emission (Fig. S2c), which might arise from contributions of Bi^3+^ orbitals in the band edges^37,46,47^. Based on theoretical calculations, Bi^3+^ incorporation is believed to modify the projected density of states at the band edges^37^, break the parity-forbidden transition of STE states^50,55^, and promote exciton localization^30^, giving rise to a new optical absorption channel at a lower energy (Fig. S3c) and promoting the PLQY of STE emission (Fig. S3a). The modified VB → CB excitation energy is then transferred to STE states responsible for visible broadband emission. Additionally, the Bi^3+^ ion has a 6s^2^ outer-electron configuration with a ^1^S_0_ ground state, and optical transitions to the 6s^1^6p^1^ configuration lead to two intense absorptions assigned to the ^1^S_0_ → ^1^P_1_ and ^1^S_0_ → ^3^P_1_-allowed transitions (inset of Fig. 2e)^54^. These two absorption transitions can effectively transfer energy to the Ln^3+^ dopants in the DP host, which are de-excited by multiple light emissions with characteristic 4f–4f transitions in the NIR spectral range. In summary, both emissions from STE recombination and Ln^3+^ 4f–4f transitions are excitable by UV light, such as light from commercial UV chips, which are beneficial for their synergistic luminescence. Indeed, as demonstrated in Fig. 2e, the PLQY for Cs_2_AgInCl_6_ DP is below 1%, Bi^3+^ doping promotes the value up to ~24% for efficient UV excitation, and Ln^3+^ codoping further increases the PLQY to 43 ~ 56% owing to the superposition of the additional Ln^3+^ emissions. Notably, the Cs_2_AgInCl_6_ DP reported by Luo et al. has been codoped with Na^+^ ions, which can significantly improve the PLQY up to ~90% by breaking the parity-forbidden transition of STE-radiative recombination^30^. Unfortunately, the doping of Na^+^ in this sample is detrimental to the incorporation of Ln^3+^ into the Cs_2_AgInCl_6_ lattice. Therefore, only the Cs_2_AgInCl_6_ DP without Na^+^ dopant was used as the host for Ln^3+^ doping.

**Fig. 3:**
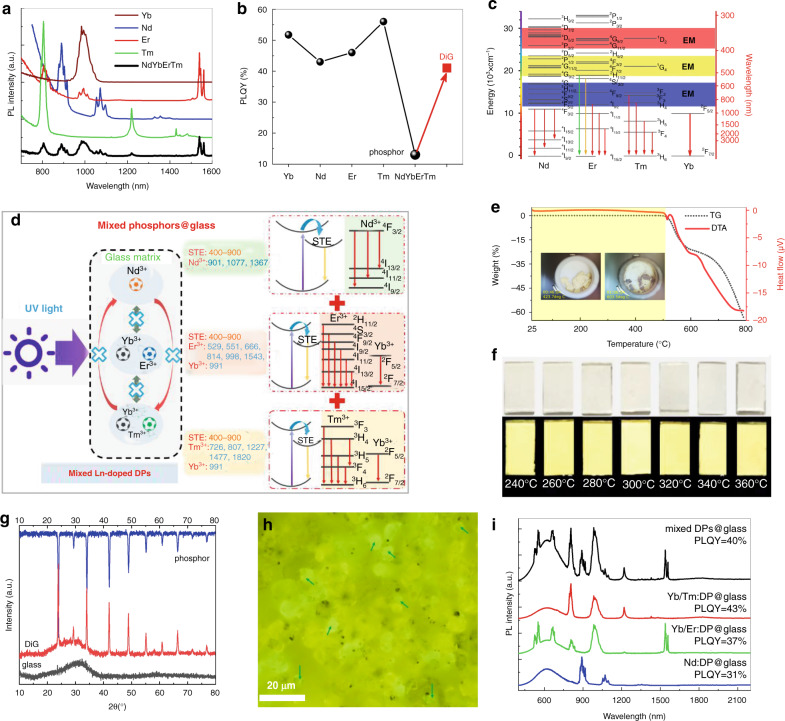
Design of “DP-in-glass” composite and optimization of the related optical performance. **a** NIR PL spectra of the Yb, Nd, Er, or Tm single-doped samples and Yb/Nd/Er/Tm multidoped samples. **b** PLQYs of the corresponding five samples and DiG composite. **c** Energy-level diagrams of the Nd, Er, Tm, and Yb activators, showing the consistent energy-migration (EM) levels (green, yellow, and blue regions), leading to coquenching PL. **d** Simplified schematic illustration of the heterogeneous architecture (DP-in-glass). **e** TG and DTA curves of a typical Bi/Er-doped sample. The insets are the samples sintered at 425 °C and 600 °C, respectively. **f** Photographs of Bi/Ln:Cs_2_AgInCl_6_–DiG–SA composites prepared with different sintering temperatures excited by daylight (top) and UV light (bottom). **g** XRD patterns of the DP phosphor, the blank glass, and the DiG composite. **h** Fluorescence image of DiG under UV-light excitation. **i** PL spectra of different kinds of DiGs

To achieve ultrabroadband luminescence in the visible–NIR spectral range, it is necessary to dope multiple Ln^3+^ activators into a sole DP host. First, the multi-doped Nd^3+^, Yb^3+^, Er^3+^, and Tm^3+^ DP samples were prepared. Compared with those of the single-doped samples, the NIR emission intensities of all the Ln^3+^ 4f–4f transitions in the multidoped sample decreased significantly (Fig. 3a). Indeed, the PLQY of the multidoped sample decreased to 13% (Fig. 3b). This PL coquenching phenomenon is attributed to detrimental energy transfers and migrations among Nd^3+^, Yb^3+^, Er^3+^, and Tm^3+^ dopants due to their well-matched and abundant multiplets^56^. As shown in Fig. 3c, the energy levels of Nd, Er, and Tm ions in the red, yellow, and blue regions are well matched, enabling energy migration among them to defect sites in the multidoped samples and finally leading to PL quenching. In addition, taking Ln^3+^ couples (such as Nd and Yb, Nd and Tm, Nd and Er, and Er and Tm) as typical examples, several energy transfer and cross-relaxation processes among Ln^3+^ ions are responsible for the energy loss via nonradiative relaxation (Fig. S13). All these detrimental energy-transfer processes finally quench the Ln^3+^ emissions in the multidoped samples. To address this challenge, we herein provide a strategy to spatially confine different Ln^3+^ dopants in a heterogeneous architecture (Fig. 3d), which involves the codispersion of Nd: Cs_2_AgInCl_6_, Yb/Er: Cs_2_AgInCl_6_, and Yb/Tm: Cs_2_AgInCl_6_ DPs into an inorganic glass matrix through a low-temperature cosintering technique^57,58^ to form a DP-in-glass (DiG) composite. Herein, the heterogeneous DiG architecture is not a multilayered structure but a monolithic composite with a size of 1 × 1 cm^2^ and a thickness of 75 µm. This method enables the effective avoidance of unwanted energy interactions among Ln^3+^ ions and produces intense Ln^3+^ NIR emissions for the separate doping of Nd, Er, and Tm ions in DPs. As shown in Fig. S13f–h, the decay lifetimes of the typical Nd^3+^: ^4^F_3/2_ → ^4^I_9/2_, Er^3+^: ^4^F_9/2_ → ^4^I_15/2_, and Tm^3+^: ^3^H_4_ → ^3^H_6_ transitions in the Nd/Yb/Er/Tm multidoped sample are far shorter than those in the Nd-, Yb/Er-, and Yb/Tm-doped samples. Importantly, the decay lifetimes recover after the DiG sample is formed, confirming the efficient inhibition of detrimental energy transfer among the various Ln^3+^-emitting centers.

The thermal stability of the Ln:Cs_2_AgInCl_6_ sample was characterized by differential thermal analysis (DTA) and thermogravimetric (TG) analysis (Fig. 3e). Both the DTA and TG curves show that the Ln:Cs_2_AgInCl_6_ sample was stable below 500 °C, and further increasing the temperature led to the gradual decomposition of the DP (inset of Fig. 3e, Fig. S14). In this case, it is important to choose an appropriate low-melting inorganic glass to successfully fabricate DiG composites. Herein, a sodium silicate glass with the composition of SiO_2_–Na_2_O–K_2_O–ZnO was selected because of its excellent moisture resistance and low melting temperature (500 °C). As illustrated in Fig. S15, the viscous Ln:Cs_2_AgInCl_6_ ink paste was first prepared by admixing Ln:Cs_2_AgInCl_6_ (Ln=Nd, Yb/Er, and Yb/Tm) phosphors, glass powders and organic vehicles via thorough grinding. Second, the ink paste was spined on a single-crystal sapphire (SA) substrate with high thermal conductivity (∼30 W m^-1^K^-1^) and then placed on a heating table for drying. Finally, it was sintered in a muffle furnace at 240–360 °C for a certain duration to obtain the Bi/Ln:Cs_2_AgInCl_6_–DiG–SA composite (Fig. 3f). The use of an SA substrate is beneficial for improving the thermal conductivity of the composite film, and the optimized synthesis temperature/time is 300 °C/15 min (Fig. S16). XRD patterns confirm that the added Ln:Cs_2_AgInCl_6_ DPs were retained in the glass without obvious decomposition (Fig. 3g). Fluorescence images of the composite film (Fig. 3h, Fig. S17) under UV-light excitation show the distribution of luminescent Bi/Ln:Cs_2_AgInCl_6_ DP particles inside the nonluminescent glass matrix. Notably, the Bi/Yb/Er:Cs_2_AgInCl_6_ microcrystals can be well distinguished from the Bi/Nd:DP and Bi/Yb/Tm:DP microcrystals for their light-green luminescence (indicated by the arrows) owing to the superimposed Er^3+^ emissions on the STE broadband emission (Fig. S17). As shown in Fig. 3i, the PLQY of the mixed multiple Bi/Ln:DPs in glass is comparable to those of the only one type of DP-embedded glasses, confirming the successful inhibition of adverse energy transfer by isolating different Ln^3+^ dopants in diverse DPs. As a consequence, the PLQY (~40%) of the three types of DP-(Nd:DP, Yb/Er:DP, and Yb/Er:DP) embedded glass is much higher than that of the Nd/Yb/Er/Tm multidoped DP phosphor (~13%, Fig. 3b).

**Fig. 4:**
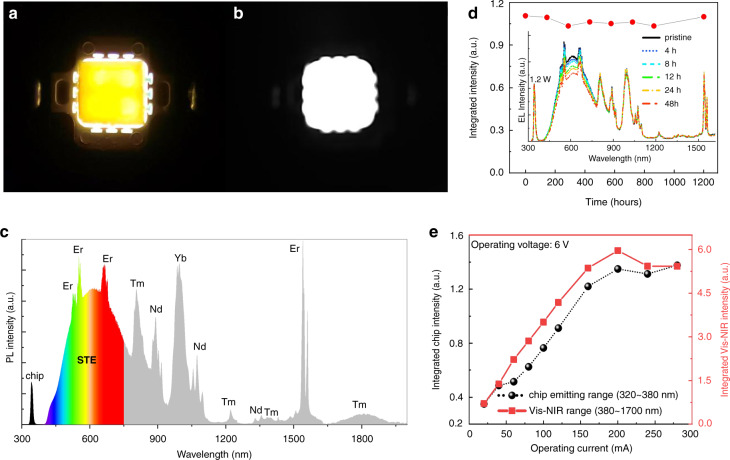
Construction of ultrabroadband LED based on Bi/Ln:DP@glass composite. Photographs of the constructed Bi/Ln:DP@glass-based LED device with an operating current of 200 mA taken by (**a**) a visible-light camera and (**b**) NIR camera. **c** PL spectrum of the LED device. **d** Dependence of the PL intensity of the LED on the exposure time in air. The inset shows the PL spectra of LEDs continuously operating for 48 h. **e** Dependence of the UV-emitting intensity of the chip and Vis–NIR-emitting intensity of DiP on the operating current from 20 to 350 mA

In a further work, a light-emitting diode (LED) was fabricated by coupling the prepared DiG with a commercial 350 nm UV chip, which can produce bright-yellow light visible to the naked eye (Fig. 4a) and intense NIR light observable by a night-vision camera (Fig. 4b). The PL spectra of the operating device show one narrow UV emission assigned to the chip, a broadband visible emission assigned to STE recombination, and many narrowband emissions ascribed to the Er^3+^, Nd^3+^, Yb^3+^, and Tm^3+^ 4f–4f transitions (Fig. 4c), covering the ultrabroad spectral region from 400 to 2000 nm with a full width at half-maximum (FWHM) of ~365 nm. The NIR emission located at ~1820 nm with a decay lifetime of 6.69 ms is assigned to the Tm^3+^: ^3^F_4_ → ^3^H_6_ transition (Fig. S18). To the best of our knowledge, this is the first report on an ultrabroadband light source based on the combination of Ln^3+^ dopants and DP. Importantly, the PL intensity remained almost unchanged after the device was exposed to air for 1200 h (Fig. 4d), confirming its super long-term stability. In fact, the DiG composite is stable after exposure to air for 180 days (Fig. S19). Moreover, after continuous operation for 48 h, only slight thermal quenching for STE emission occurred, and no obvious changes in the Ln^3+^ emissions were observed in the PL spectra (inset of Fig. 4d). The STE emission is sensitive to temperature (Fig. S20a), and the increased temperature of the UC chip during operation leads to a certain degree of PL quenching. Additionally, the 4f orbitals of the Ln^3+^ ions are shielded from the surroundings by the filled 5s^2^ and 5p^6^ orbitals. Therefore, the influence of temperature on the optical transitions within the 4f^n^ configuration is small since there is no crossover point between the excited state and the ground state in the Ln^3+^ configuration^13^. In this case, no obvious change in the Ln^3+^ PL intensity was detected with increasing temperature, while PL quenching of STE was observed since exciton recombination is highly sensitive to temperature (Fig. S20b). Fortunately, the temperature of the constructed u-LED with long-term operation is stable (remained below 30 °C) owing to the use of a cooling fan (Fig. S21). Therefore, thermal quenching only subtly impacted the u-LED performance after several weeks. The excellent stability of the device is ascribed to the effective protection of the inorganic glass matrix for the Bi/Ln:Cs_2_AgInCl_6_ color converters, which inhibits the decomposition of and damage to the DPs upon exposure to external stimuli. As shown in Fig. 4e, both the UV and NIR emissions increased monotonically with increasing operating current from 20 mA to 200 mA and tended to be stable for higher input currents owing to the saturation of the commercial 350 nm UV chip.

**Fig. 5:**
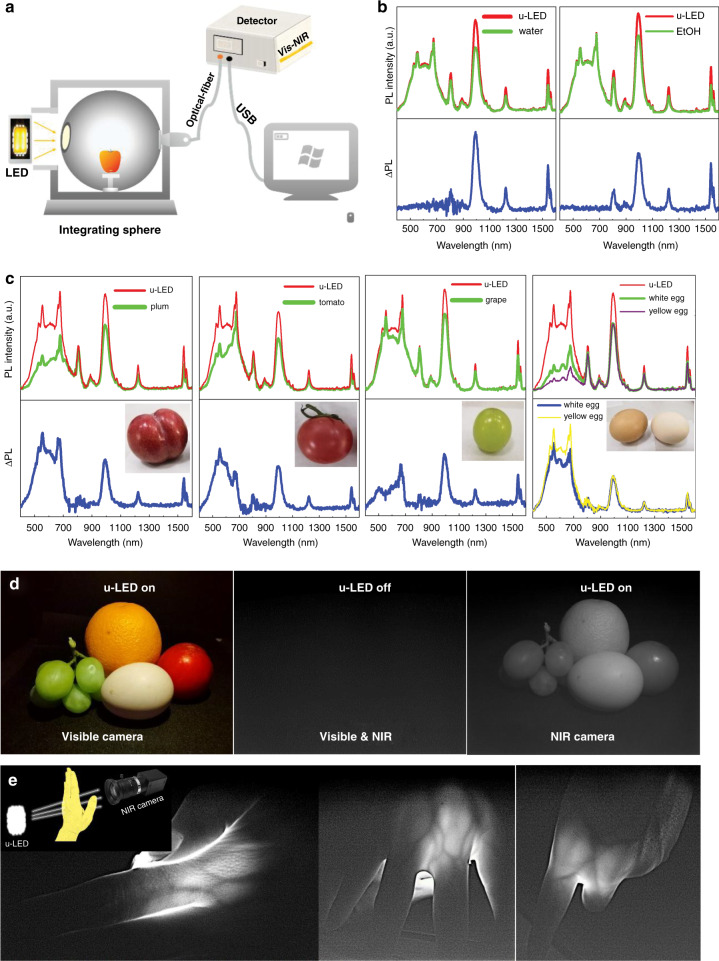
Application demonstration of Bi/Ln:DP-based ultrabroadband LED. **a** Schematic diagram of the experimental setup for measuring absorption. PL spectra with and without the measured substance inside the integrated sphere using u-LED as the light source: (**b**) water and EtOH, (**c**) plum, tomato, grape, and eggs. ΔPL is defined as PL(u-LED) - PL(u-LED + substance). **d** Photos of fruits with and without u-LED irradiation recorded by visible and NIR cameras. **e** Photos of palm and finger after transillumination of NIR light from u-LED

Finally, we demonstrate the promising applications of this compact ultrabroadband LED (u-LED) in nondestructive spectroscopic analysis and multifunctional lighting. The absorption spectra of some substances, such as water, ethyl alcohol (EtOH), plum, tomato, grape, and white/yellow egg, were measured using the u-LED as the light source. The sample was placed inside an integrating sphere equipped with the u-LED and a fiber-coupled spectrometer (Fig. 5a). The absorption spectrum of the sample was obtained by subtracting the emission spectrum of the u-LED recorded without the sample from that recorded with the sample. As shown in Fig. 5b, the absorption of water and EtOH in the visible spectral region is negligible, and several NIR-absorption peaks located at 810 nm, 994 nm, 1220 nm, and 1545 nm are observed. In comparison, the fruits and eggs showed strong absorption in the visible spectral range in addition to NIR-absorption peaks; these absorption signals were similar to those observed in water and EtOH (Fig. 5c). The visible absorption intensity of dark-red plum, red tomato, and green grape decreases in sequence, which is probably due to the relationship between the color of fruits and the absorption of visible light by phytochromes. A similar phenomenon can be observed for eggs with different apparent colors (white and red), where the visible absorption of white eggs is remarkably lower than that of red eggs. The exact origin of absorption is not discussed, and quantitative analysis is not performed in the present work due to its complexity and because it is beyond the scope of this research. Figure 5d and Fig. S22 present photographs of fruits and palms taken via different cameras (visible camera and NIR camera). No image can be captured by either camera when the u-LED is off (middle of Fig. 5d). In contrast, when the u-LED lamp is lightened, the colorful image (left of Fig. 5d, Fig. S22a) and black-and-white image (right of Fig. 5d, Fig. S22b) are easily detected by the visible camera and NIR camera, respectively. This kind of dual-modal lighting based on u-LED is ascribed to its broadband emissions covering both visible and NIR spectral regions. In addition, when the light from the u-LED is passed through the palm and fingers, the venous blood vessels in the palm and fingers are discerned by the NIR camera (Fig. 5e, Fig. S22) owing to the loss of NIR light absorbed by the chromophores in the blood.

## Conclusions

In summary, a family of Ln^3+^ ions and Bi^3+^ ions was successfully doped into Cs_2_AgInCl_6_ DP microcrystals prepared by a modified coprecipitation method. Bi^3+^ ions were easily incorporated into DPs by substituting In^3+^, while Ln^3+^ doping resulted in a saturation concentration of ~2.5 mol%. Bi^3+^ doping can decrease the excitation energy to the UV region (330–400 nm) for STE recombination and significantly promote the PLQY from ~1% to ~25% owing to the Bi^3+^-doping-induced modification of the band-edge structure of Cs_2_AgInCl_6_ DP. Importantly, Ln^3+^ codoping can further increase the PLQY to 43~56% owing to the superimposition of the additional Ln^3+^ emissions on the broadband STE luminescence. A series of spectroscopic characterizations evidence the different energy-transfer channels for STE emission and Ln^3+^ 4f–4f emissions, where the former is attributed to energy transfer from band-edge to STE state and the latter is due to energy transfer from Bi^3+^ in the 6s^1^6p^1^ state to the Ln^3+^ multiplets. In this case, Nd/Yb/Er/Tm multidoping in Bi:DP can produce ultrabroadband luminescence covering the Vis and NIR spectral regions. Furthermore, a DiG composite was fabricated by dispersing diverse Nd:DP, Yb/Er:DP, and Yb/TmDP in an inorganic glass matrix to spatially confine the various Ln^3+^ dopants, which is beneficial for inhibiting the coquenching PL for multiple Ln^3+^ ions in a single DP host. Finally, the DiP-based light source was designed; it exhibited a commercial UV chip with excitable ultrabroadband (400 ~ 2000 nm) electroluminescence. This compact u-LED has a low cost, is easy to manufacture, is nontoxic, exhibits excellent optoelectronic performance, and is stable; therefore, it provides practicality, commercialization, and industrialization in multifunctional lighting and nondestructive spectral analysis.

## Methods

### Chemicals

Indium(III) trichloride (InCl_3_, Shanghai Macklin Chem. Co., LTD., 99.9%), bismuth trichloride (BiCl_3_, Macklin, 99.9%), silver chloride (AgCl, Macklin, 99.5%), hydrochloric acid (HCl, Hushi, 99.99%), cesium chloride (CsCl, Macklin, 99.9%), and lanthanide(III) chloride hexahydrate (LnCl_3_·6H_2_O, Ln=La–Lu, Beijing HWRK Chem. Co., LTD., 99.99%). All of the chemicals were used without further purification.

### Synthesis of Bi^3+^-doped Cs_2_AgInCl_6_

Before the reaction, a shaded and heat-resistant glass bottle was prepared in advance to prevent the oxidation of silver chloride (movie 1). First, different amounts of BiCl_3_ (0, 0.003, 0.006, 0.012, 0.024, 0.036, 0.048, 0.060, and 0.100 mmol), 0.25 mmol of InCl_3_, 0.25 mmol of AgCl, and 4 mmol of 12 M HCl were added to an 8 mL glass bottle. Second, the glass bottle was placed in an oil bath pan at 100 °C and stirred vigorously, until all the powders in the reaction mixture were completely dissolved. Third, 0.5 mmol CsCl was dissolved in 1 mL of 12 M HCl and swiftly injected into the glass bottle to produce the products. The reaction continued for another 20 min to ensure that it was complete. Then, the glass bottle was magnetically stirred at room temperature for 10 min. The crude microcrystalline solution was centrifuged at 10000 rpm for 5 min, and the supernatant was discarded. The residual sediment was washed twice with ethanol, and the solution was then centrifuged at 10000 rpm for 10 min. Finally, the precipitates were dried at 80 °C in a drying oven overnight and then stored.

### Synthesis of Bi/Er codoped Cs_2_AgInCl_6_

The synthesis process was similar to that of Bi^3+^-doped Cs_2_AgInCl_6_. The only difference was that the BiCl_3_ content was fixed at 0.048 mmol, and different amounts of ErCl_3_ (0, 0.25, 0.50, 0.75, 1.00, and 1.25 mmol) were added.

### Synthesis of Bi/Ln (Ln=La–Lu) codoped Cs_2_AgInCl_6_

The synthesis process was similar to that of Bi^3+^-doped Cs_2_AgInCl_6_. The only difference was that the BiCl_3_ and LnCl_3_ contents were fixed at 0.048 mmol and 0.75 mmol, respectively.

### Synthesis of Bi/Yb/Ln (Ln=Er, Tm, or Ho) triple-doped Cs_2_AgInCl_6_

The synthesis process was similar to that of Bi^3+^-doped Cs_2_AgInCl_6_. The only difference was that the BiCl_3_, YbCl_3_, and LnCl_3_ (Ln=Er^3+^, Tm^3+^ or Ho^3+^) contents were fixed at 0.048 mmol, 0.75 mmol and 0.75 mmol, respectively.

### Synthesis of Bi/Nd/Yb/Er/Tm multidoped Cs_2_AgInCl_6_

The synthesis process was similar to that of Bi^3+^-doped Cs_2_AgInCl_6_. The only difference was that the BiCl_3_ content was fixed at 0.048 mmol and all LnCl_3_ contents were fixed at 0.75 mmol.

### Fabrication of Bi/Ln:Cs_2_AgInCl_6_@glass

First, diethylene glycol monobutyl ether acetate (DBAC), ethyl cellulose (EC), and terpineol precursors were added to a glass bottle. Then, the slurry was heated to 80 °C for at least 50 min to fully react. In addition, the inorganic glass frits with compositions of SiO_2_–Na_2_O–K_2_O–ZnO and the Bi-/Ln-doped DP phosphors were ground with a small amount of the above-mentioned slurry for 20 min and then spined on the single-crystal sapphire. Finally, the composite film was sintered in a muffle furnace at 240–360 °C for 15/30 min to obtain DP-in-glass (DiG). Notably, for ultrabroadband LED applications, DiG is a monolithic composite, where Nd:Cs_2_AgInCl_6_, Yb/Er:Cs_2_AgInCl_6_, and Yb/Tm:Cs_2_AgInCl_6_ DPs are codispersed inside an inorganic glass matrix.

### Fabrication of DiG-based LEDs

The compact ultrabroadband-emitting light-emitting diode (u-LED) was constructed by coupling the prepared monolithic DiG layer with a commercial 350 nm UV chip by placing the DiG on the surface of the UV chip. A cooling fan was adhered to the back of the UV chip to reduce the thermal accumulation upon operation. The opaque silica gels were filled around the edges between DiG and the UV chip to prevent the leakage of UV light.

### Characterizations

X-ray diffraction (XRD) analysis was carried out to identify Bi/Ln:Cs_2_AgInCl_6_ crystalline-phase structures using a Bruker D8 Advance X-ray powder diffractometer with Cu K_α_ radiation (*λ* = 0.154 nm) operating at 40 kV. The actual chemical compositions were measured by inductively coupled plasma–mass spectrometry (ICP–MS) using a Perkins-Elmer Optima 3300DV spectrometer. The microstructures of the samples were characterized by scanning electron microscopy (SEM) equipped with an energy-dispersive X-ray (EDX) spectroscopy system and were visualized via fluorescence images using optical microscopy (Nikon, Eclipse, Ti2) coupled with a spectrofluorometer. The elemental analysis of Bi/Ln:Cs_2_AgInCl_6_ was performed by X-ray photoelectron spectroscopy (XPS) using a VG Scientific ESCA Lab Mark II spectrometer equipped with two ultrahigh vacuum-6 (UHV) chambers. All the binding energies were referenced to the C_1s_ peak of the adventitious carbon on the surface at 284.8 eV. ^133^Cs and ^115^In magic-angle-spinning nuclear magnetic resonance (MAS-NMR) spectra were recorded using Bruker ADVANCE III HD 400 spectrometers. The diffuse-reflectance spectra were recorded on a spectrophotometer (Lambda900, Perkin-Elmer) with a resolution of 1 nm. Thermogravimetric analyses (TGA) were conducted on a Netzsch STA449C thermal analysis system under a N_2_ flow with a heating rate of 10 °C min^−1^. Photoluminescence (PL) spectra, PL-excitation (PLE) spectra and decay curves were recorded on an Edinburgh Instruments FLS1000 spectrofluorometer equipped with a continuous xenon lamp (450 W), a pulsed flash lamp, and a 375 nm picosecond pulsed laser. Middle-infrared (MIR) PL emission spectra and decay curves were measured with a customized UV to mid-infrared steady-state and phosphorescence-lifetime spectrometer (FSP920-C, Edinburgh) equipped with a tuneable midband optical parametric oscillator pulsed laser as the excitation source (210–2400 nm, 10 Hz, pulse width ≤5 ns, Opolette^TM^ HE355UV, OPOTEK). The absolute photoluminescence quantum yields (PLQYs) of the samples were obtained by employing a standard barium sulfate-coated integrating sphere (150 mm in diameter, Edinburgh) as the sample chamber that was mounted on the FLS1000 spectrometer with the entry and output port of the sphere located at a 90° angle from each other in the plane of the spectrometer. A standard tungsten lamp was used to correct the optical response of the instrument. All the spectral data were collected at RT and corrected for the spectral response of both the spectrometer and the integrating sphere. PLQY_(450–1600 nm)_ of Ln:DP covering the Vis–NIR broad spectral range equals the sum of PLQY_(450–930 nm)_ and PLQY_(930–1600 nm)_. PLQY_(930–1600 nm)_ was determined by the expression PLQY_(650–930 nm)_ × I_(930–1600 nm)_/I_(650–930 nm)_. Note that the integral intensities for both I_(930–1600 nm)_ and I_(650–930 nm)_ here come from the same near-infrared spectra (620–1650 nm). PL spectra of the UV LED chip and the fabricated u-LED were measured using a Keithley 2400 source and an integrating sphere equipped with an FLS1000 spectrofluorometer.

## Supplementary information

Supplementary information

Movie 1
