# Supplementary material for: Compact ultrabroadband light-emitting diodes based on lanthanide-doped lead-free double perovskites
Source: Light Sci Appl. 2022 Mar 8;11:52. doi: 10.1038/s41377-022-00739-2 (PMC8901751; doi:10.1038/s41377-022-00739-2)
Supplement: Supplementary file 1 — Supplementary information [file 41377_2022_739_MOESM1_ESM.pdf]

Supplementary Information for

Compact ultra-broadband light-emitting diodes based on lanthanide-doped lead-free double perovskites

Shilin Jin [a, b], Renfu Li [c, *], Hai Huang [a], Naizhong Jiang [a], Jidong Lin [a], Shaoxiong Wang [a, d, e], Yuanhui Zheng [b, f], Xueyuan Chen [c, *], Daqin Chen [a, b *]

[a] College of Physics and Energy, Fujian Normal University, Fujian Provincial Key Laboratory of Quantum Manipulation and New Energy Materials, Fuzhou, 350117, China
E-Mail: dqchen@fjnu.edu.cn (D. Q. Chen)

[b] Fujian Science & Technology Innovation Laboratory for Optoelectronic Information, Fuzhou, 350116, China

[c] CAS Key Laboratory of Design and Assembly of Functional Nanostructures, Fujian Key Laboratory of Nanomaterials and State Key Laboratory of Structural Chemistry, Fujian Institute of Research on the Structure of Matter, Chinese Academy of Sciences, Fuzhou, Fujian 350002, China
E-Mail: lirenfu@fjirsm.ac.cn (R. F. Li); xchen@fjirsm.ac.cn (X. Y. Chen)

[d] Fujian Provincial Collaborative Innovation Center for Advanced High-Field Superconducting Materials and Engineering, Fuzhou, 350117, China

[e] Fujian Provincial Engineering Technology Research Center of Solar Energy Conversion and Energy Storage, Fuzhou, 350117, China

[f] College of Chemistry, Fuzhou University, Fuzhou, 350116, China

Table S1. The tabulation of a series of Ln^{3+} doped DPs and their bandgap types, preparation methods, PL ranges, PLQYs and possible applications. NCs, MCs, SC represent nanocrystals, microcrystals and single crystal. FTM, NIR-LED, pc-wLED represent falling temperature method, near-infrared light-emitting diode, and phosphor-converted white light-emitting diode, respectively.

Material	Dopant	Bandgap type	Method	PL (nm)	PLQY (%)	Application	Ref.
$Cs_2NaBiCl_6$ NCs	Eu	Indirect	Hot injection	591, 615	3.00	/	S1
$Cs_2AgBiBr_6$ MCs	Eu/Yb	Indirect	Hydrothermal	/	28.00	/	S2
Cs_2AgBiX_6 NCs	Yb	Indirect	Hot injection	994	/	/	S3
$Cs_2NaTbCl_6$ SC	Ce	Indirect	FTM	386	/	Scintillator	S4
$Cs_2NaScCl_6$ SC	Sc	Indirect	FTM	400-550	29.05	/	S5
$Cs_2Ag_{0.6}Na_{0.4}InCl_6$ SC	Bi/Yb/Er	Direct	FTM	527, 555, 660	/	X-ray Detection, Anti-Counterfeiting	S6
$Cs_2Na_{0.6}Ag_{0.4}InCl_6$ MCs	Bi/Yb	Direct	Solvothermal	400-800, 996	84.70	NIR-LED	S7
$Cs_2Ag_{0.4}Na_{0.6}InCl_6$ MCs	Bi/Ce	Direct	Solvothermal	400-800	89.90	pc-wLED	S8
$Cs_2Ag_{0.6}Na_{0.4}InCl_6$ MCs	Bi/Yb	Direct	Solvothermal	400-1050	70.30	Temperature Sensor	S9
$Cs_2(Na,Ag)InCl_6$ MCs	Bi/Ho	Direct	FTM	430~800	57.09	pc-wLED	S10
$Cs_2AgInCl_6$ NCs	Yb/Er	Direct	Solvothermal	996, 1537	3.60, 0.05	/	S11
$Cs_2AgInCl_6$ NCs	Bi/La	Direct	Hot injection	400-800	60	/	S12
$Cs_2AgInCl_6$ NCs	Bi/Tb	Direct	Hot injection	490, 550, 620	/	/	S13
$Cs_2AgInCl_6$ MCs	Bi/Yb/Er	Direct	Solvothermal	1540, 994	/	NIR-LED	S14
$Cs_2AgInCl_6$ NCs/MCs	Yb	Direct	Solvothermal	994	/	/	S15

Table S2. Comparison of dopant concentrations obtained from ICP-MS data for the Bi: $Cs_2AgInCl_6$, Bi/Er: $Cs_2AgInCl_6$ and Bi/Yb/Er: $Cs_2AgInCl_6$ products. All the samples have fixed Bi nominal content of 0.048 mmol and various Ln^{3+} doping contents. The nominal ratio relative to In^{3+} ion and the actual doping content are determined based on the equations of [X]/[In] and [X]/[X+In], respectively.

**Bi/Ln: $Cs_2AgInCl_6$**	**Nominal ratio**			**Actual content (ICP-MS)**		
	Bi/In	Er/In	Yb/In	Bi^{3+} (%)	Er^{3+} (%)	Yb^{3+} (%)
Er (0.00 mmol)	0.192	/	/	14.60	/	/
Er (0.25 mmol)	0.192	1	/	/	0.56	/
Er (0.50 mmol)	0.192	2	/	/	2.26	/
Er (0.75 mmol)	0.192	3	/	14.79	2.40	/
Er (1.00 mmol)	0.192	4	/	/	2.43	/
Er (1.25 mmol)	0.192	5	/	/	2.55	/
Er/Yb (0.75/0.75 mmol)	0.192	3	3	14.76	1.12	1.29

Table S3. Elemental percentage (mol%) in the Bi/Yb/Er: $Cs_2AgInCl_6$ sample determined from EDX data.

**Element**	**Cs**	**Ag**	**In**	**Cl**	**Bi**	**Er**	**Yb**
Content (mol%)	19.65	8.27	7.81	62.11	1.06	0.14	0.17

Table S4. The tabulation of diverse Ln^{3+} 4f→4f transitions and emitting wavelength ranges for the Bi/Ln: $Cs_2AgInCl_6$ samples determined from the recorded PL spectra.

Ln^{3+}	4f→4f Transition / emitting wavelength range (nm)					
Pr^{3+}	$^3P_1 \to {}^3F_3$	$^1G_4 \to {}^3H_4$	$^1D_2 \to {}^3F_4$			
	643~666	983~1011	1025~1082			
Nd^{3+}	$^4F_{3/2} \to {}^4I_{9/2}$	$^4F_{3/2} \to {}^4I_{11/2}$	$^4F_{3/2} \to {}^4I_{13/2}$			
	871~931	1045~1110	1323~1411			
Sm^{3+}	$^4G_{5/2} \to {}^6H_{5/2}$	$^4G_{5/2} \to {}^6H_{7/2}$				
	560~580	593~608				
Tb^{3+}	$^5D_4 \to {}^7F_5$					
	541~555					
Dy^{3+}	$^4F_{9/2} \to {}^6H_{13/2}$	$^6F_{3/2} \to {}^6H_{15/2}$	$^6H_{9/2} \to {}^6H_{15/2}$			
	573~587	749~760	1282~1378			
Ho^{3+}	$^5F_3 \to {}^5I_8$	$^5S_2 \to {}^5I_8$	$^5F_5 \to {}^5I_8$	$^5I_5 \to {}^5I_8$	$^5I_6 \to {}^5I_8$	
	478~500	539~561	638~674	962~1004	1170~1201	
Er^{3+}	$^2H_{11/2} \to {}^4I_{15/2}$	$^4S_{3/2} \to {}^4I_{15/2}$	$^4F_{9/2} \to {}^4I_{15/2}$	$^4I_{9/2} \to {}^4I_{15/2}$	$^4I_{11/2} \to {}^4I_{15/2}$	$^4I_{13/2} \to {}^4I_{15/2}$
	521~539	542~565	653~687	793~838	973~1022	1531~1567
Tm^{3+}	$^3F_3 \to {}^3H_6$	$^3H_4 \to {}^3H_6$	$^3H_5 \to {}^3H_6$	$^3H_4 \to {}^3F_4$		
	684~717	783~830	1193~1261	1427~1523		
Yb^{3+}	$^2F_{5/2} \to {}^2F_{7/2}$					
	981~1007					

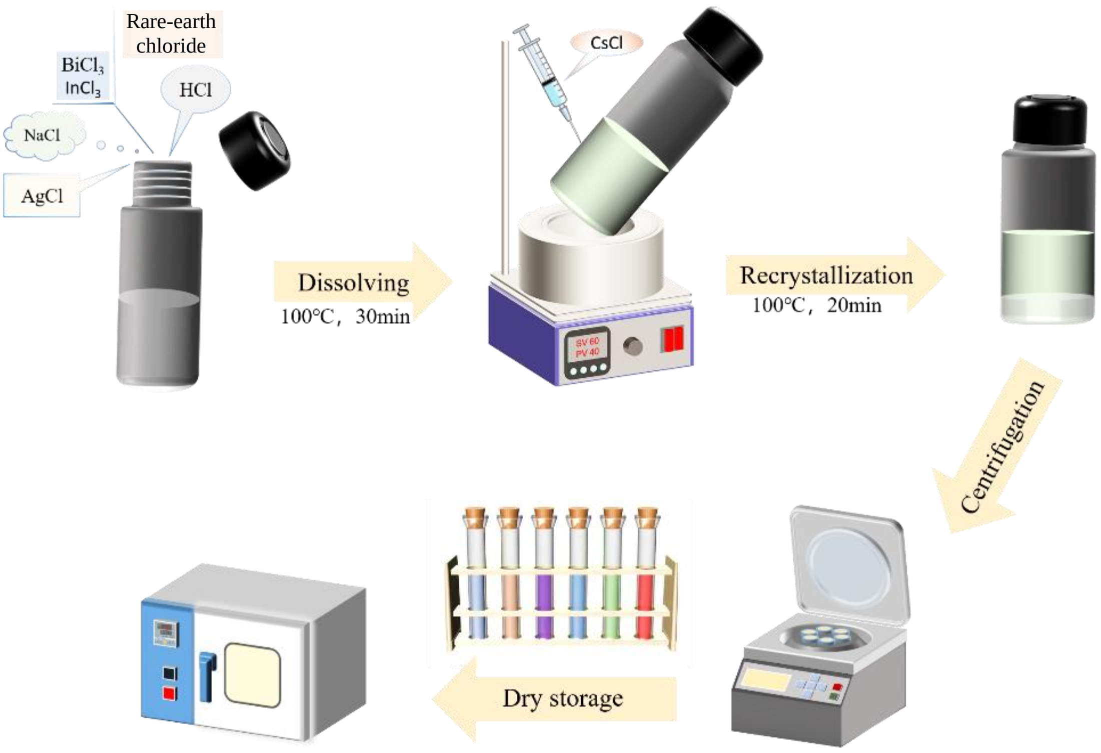

Figure S1. A schematic procedure for preparing Bi/Ln doped $Cs_2AgInCl_6$ sample.

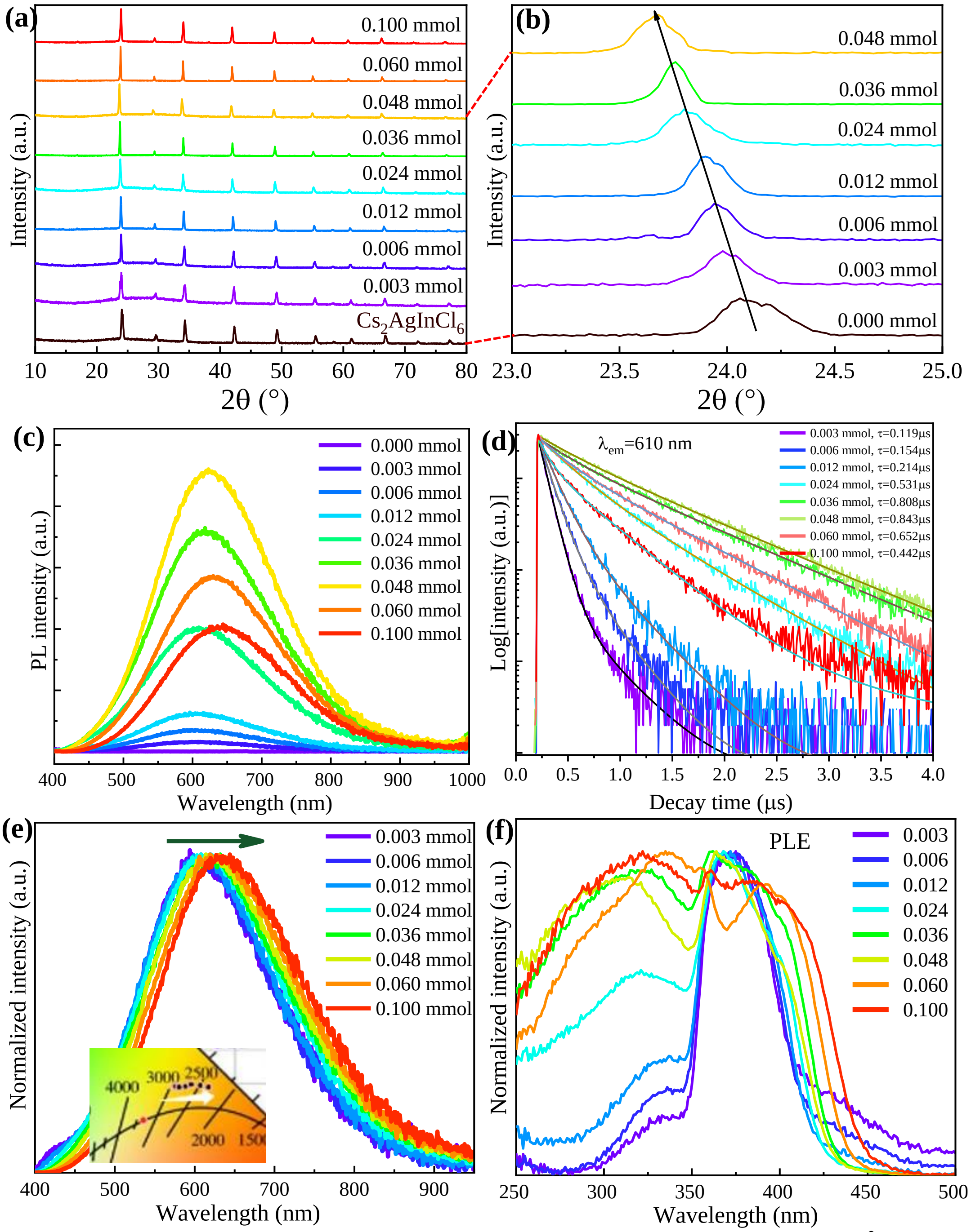

Figure S2. a) XRD patterns of Bi-doped $Cs_2AgInCl_6$ samples with different Bi^{3+} contents. b) Enlarged XRD patterns at a certain diffraction peak for Bi-doped $Cs_2AgInCl_6$ samples with Bi contents of 0~0.048 mmol. c) PL spectra and d) PL decay curves by monitoring STE emission (λ_{em}=610 nm) for the Bi-doped $Cs_2AgInCl_6$ samples. e) Normalized PL and f) PLE spectra for the corresponding Bi-doped samples. Inset of e) is the color coordinates of PL spectra in CIE diagram.

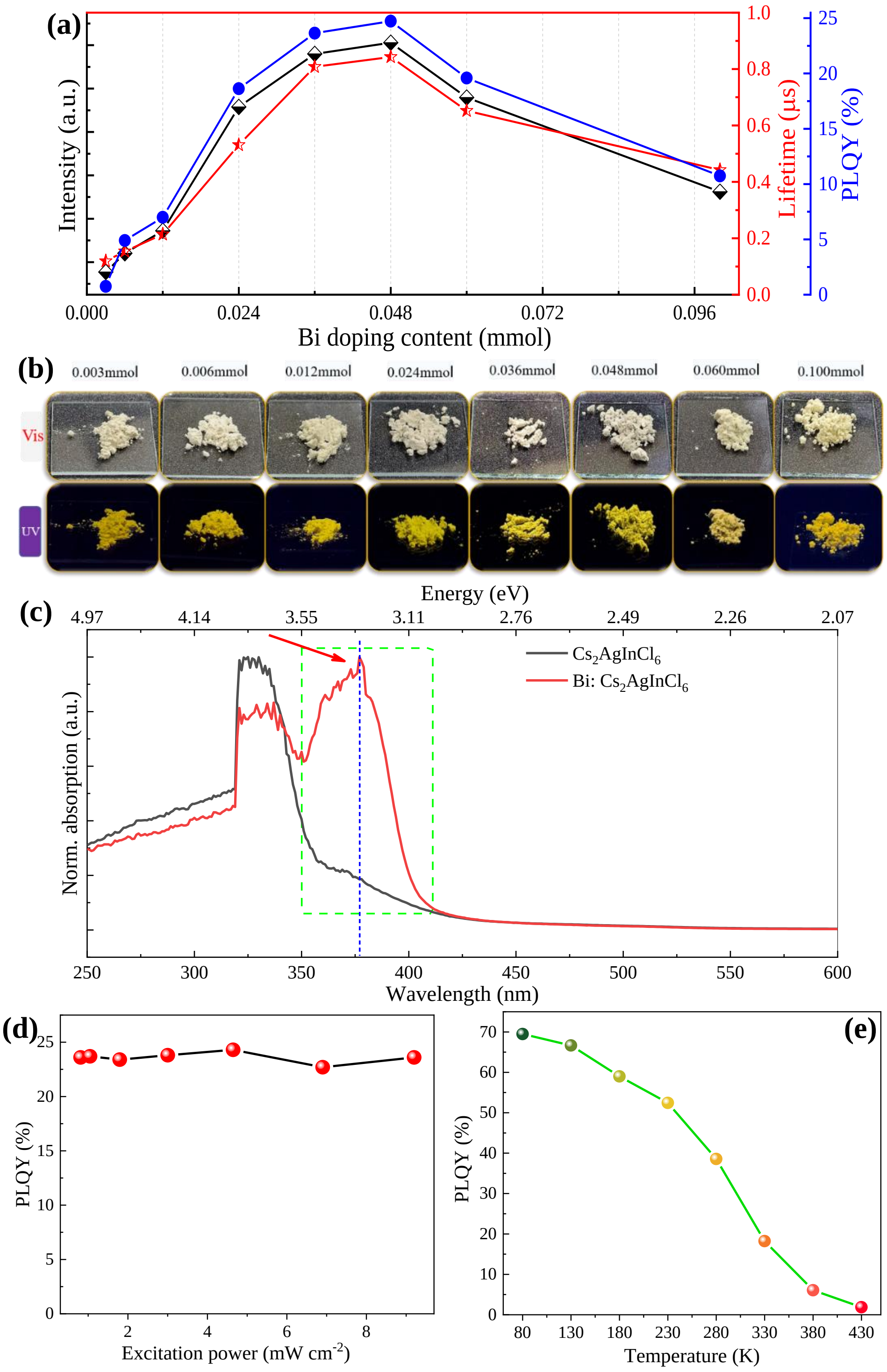

Figure S3. a) Dependence of integrated intensity, lifetime and PLQY for STE emission on the Bi doping content. b) The corresponding samples under daylight and UV light excitation. c) Comparison of absorption spectra for the $Cs_2AgInCl_6$ and Bi: $Cs_2AgInCl_6$ samples. d) Excitation power dependent PLQYs at room temperature and e) temperature dependent PLQYs for the Bi: $Cs_2AgInCl_6$ sample.

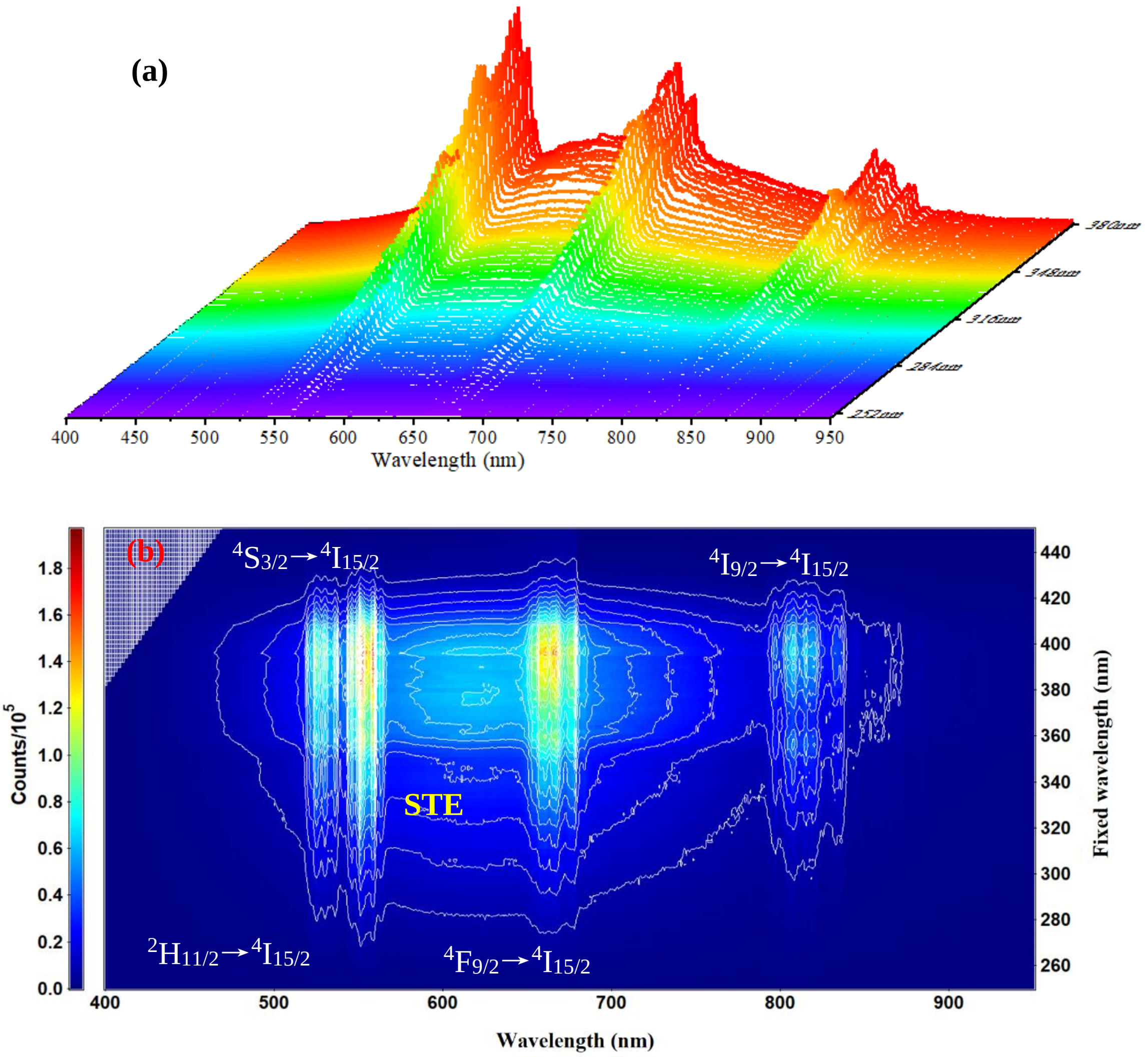

Figure S4. Dependence of PL spectra for the Bi/Er: $Cs_2AgInCl_6$ sample on the excitation wavelength (250-450 nm): a) the 3D plot and b) the contour plot.

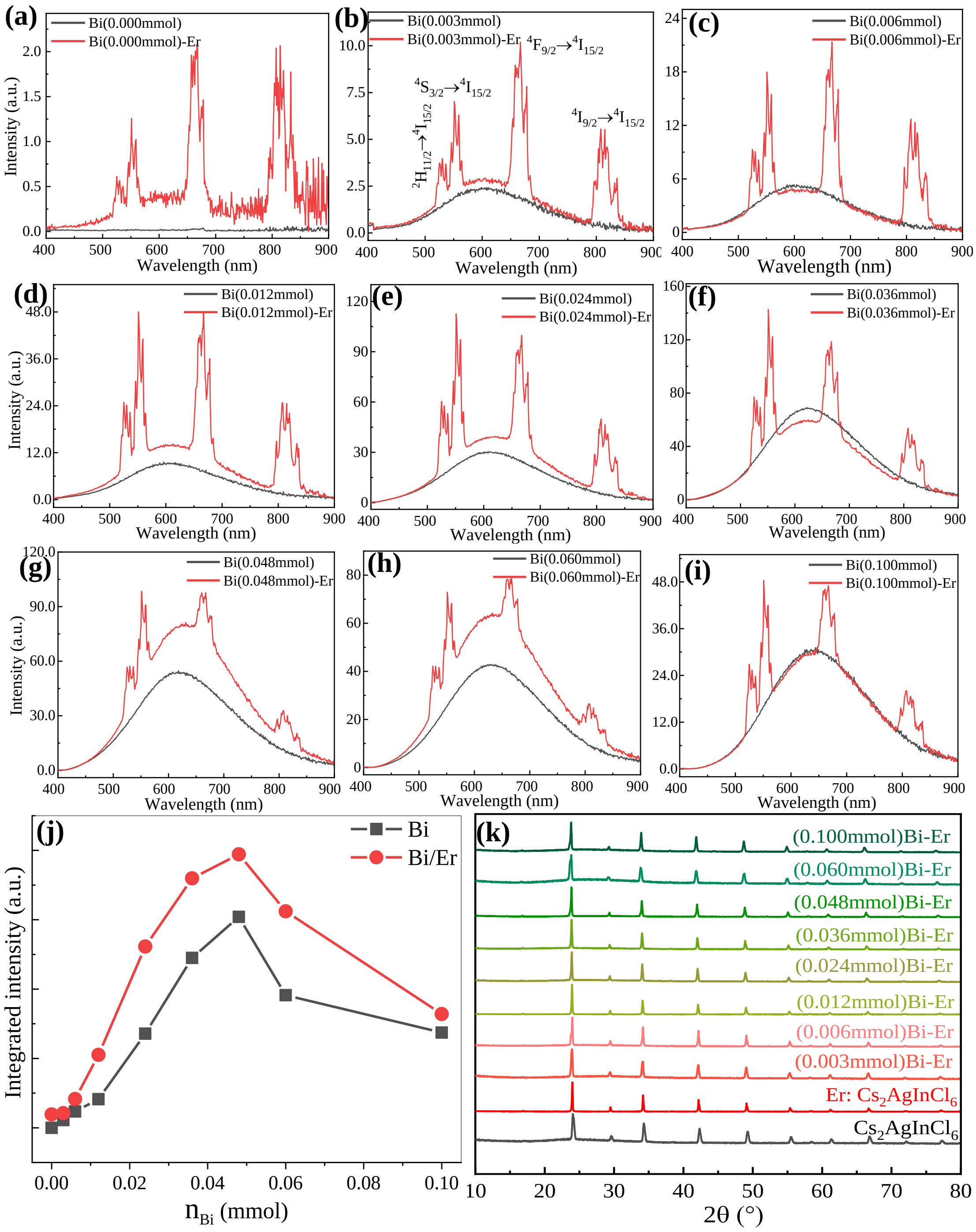

Figure S5. a-i) PL spectra of Bi-doped and Bi/Er co-doped $Cs_2AgInCl_6$ samples with fixed Er nominal content (0.75 mmol) and diverse Bi nominal contents (0~0.1 mmol) recorded under the excitation of 350 nm UV light. j) Dependence of PL integrated intensities of Bi-doped and Bi/Er co-doped $Cs_2AgInCl_6$ samples on Bi doping content. k) XRD patterns of the undoped, Er-doped and Bi/Er co-doped $Cs_2AgInCl_6$ samples.

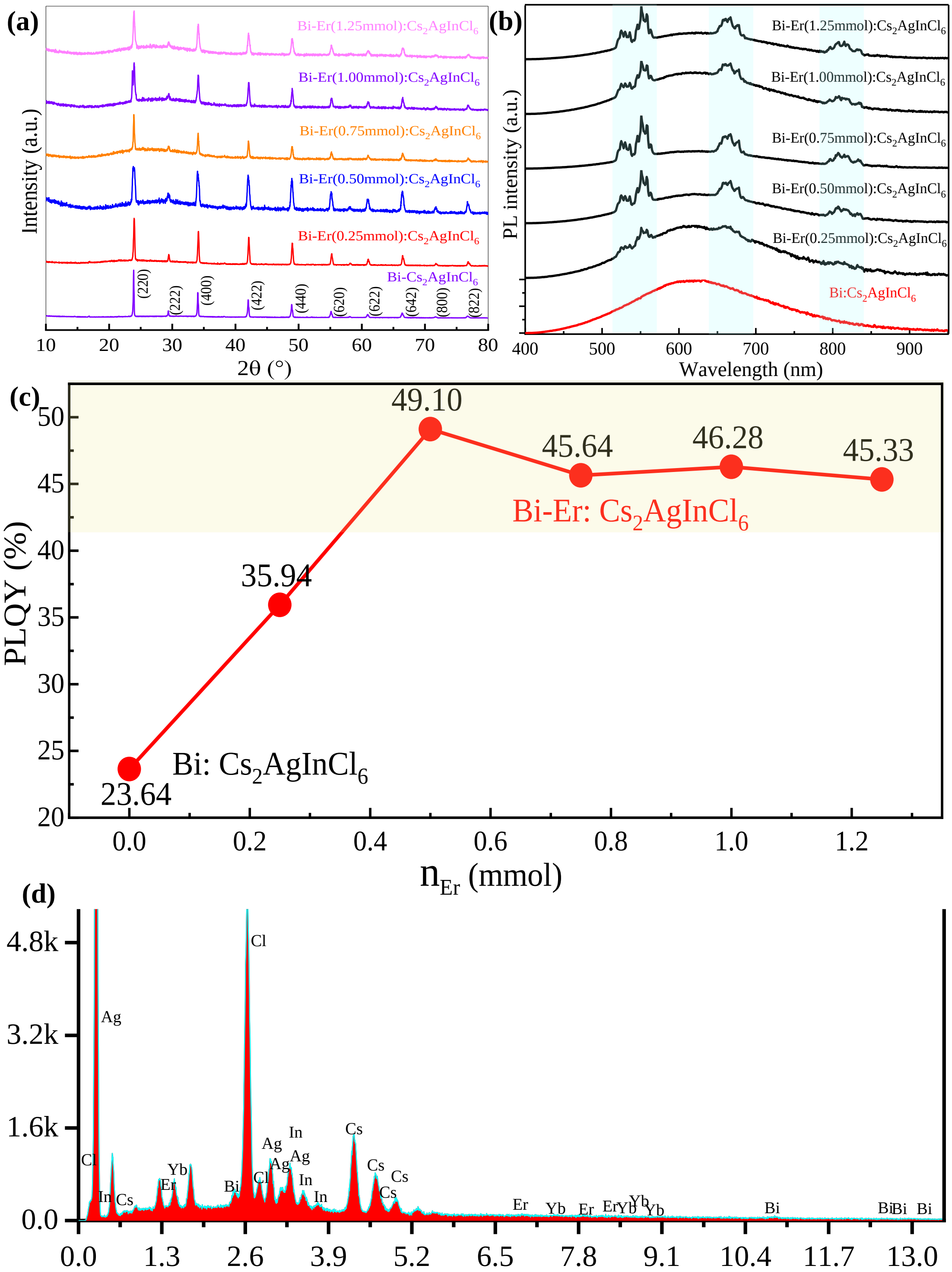

Figure S6. a) XRD patterns and b) PL spectra of Bi/Er: $Cs_2AgInCl_6$ samples with the fixed Bi content and various Er doping contents. c) PLQY of the sample versus Er^{3+} nominal doping content. d) EDX spectrum recorded from the Bi/Yb/Er: $Cs_2AgInCl_6$ sample, showing the existence of Bi, Yb, Er elemental signals.

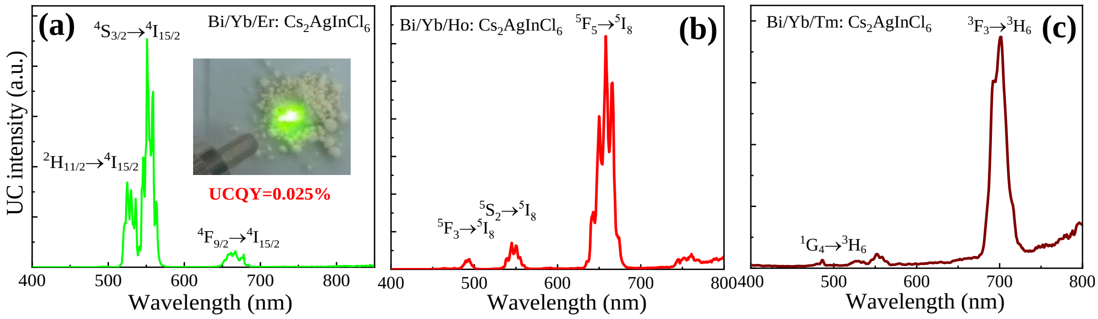

Figure S7. Upconversion (UC) emission spectra of the Bi/Yb/Ln: $Cs_2AgInCl_6$ samples under 980 nm laser excitation: a) Ln=Er, b) Ln= Ho and c) Ln= Tm. Inset is the corresponding UC emitting photograph and UCQY value of Bi/Yb/Er: $Cs_2AgInCl_6$ sample.

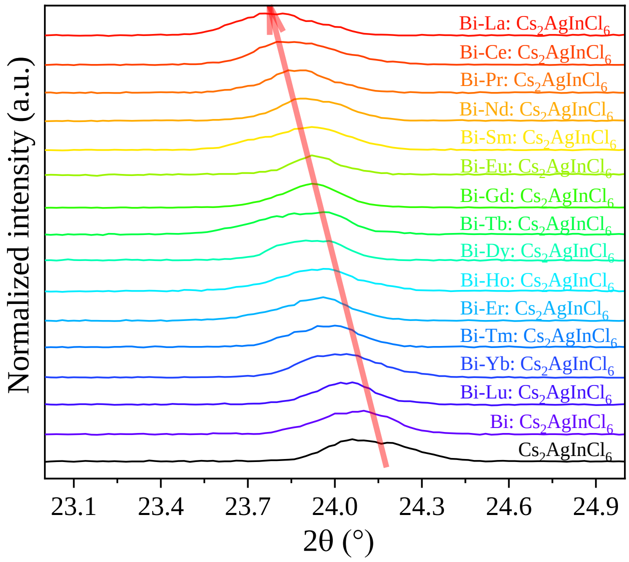

Figure S8. The enlarged view of XRD patterns with the peak at ~ 24 ^{o} for the Bi/Ln: $Cs_2AgInCl_6$ (Ln=Lu-La) DPs.

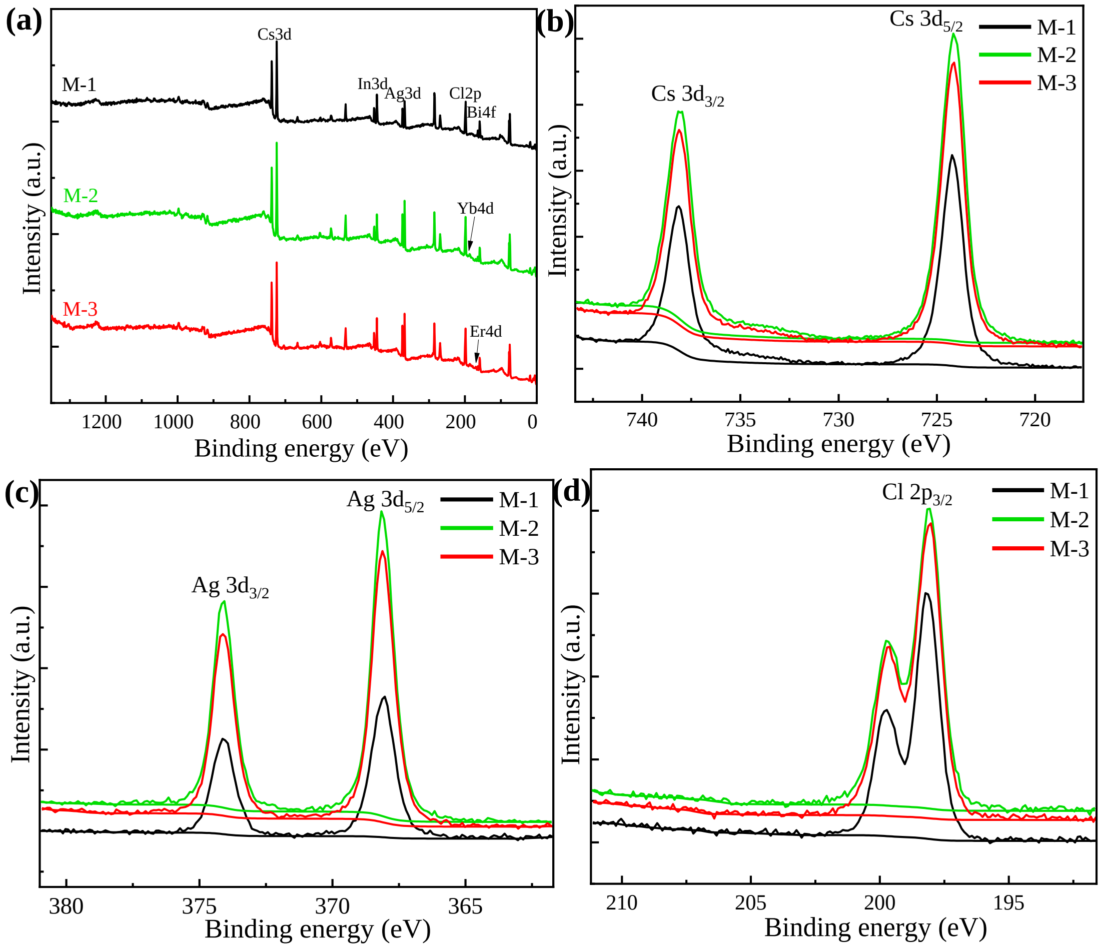

Figure S9. X-ray photoelectron spectra of a) all the elements, b) Cs 3d, c) Ag 3d and d) Cl 2p for the M-1(Bi: $Cs_2AgInCl_6$), M-2 (Bi/Yb: $Cs_2AgInCl_6$) and M-3 (Bi/Yb/Er: $Cs_2AgInCl_6$) samples.

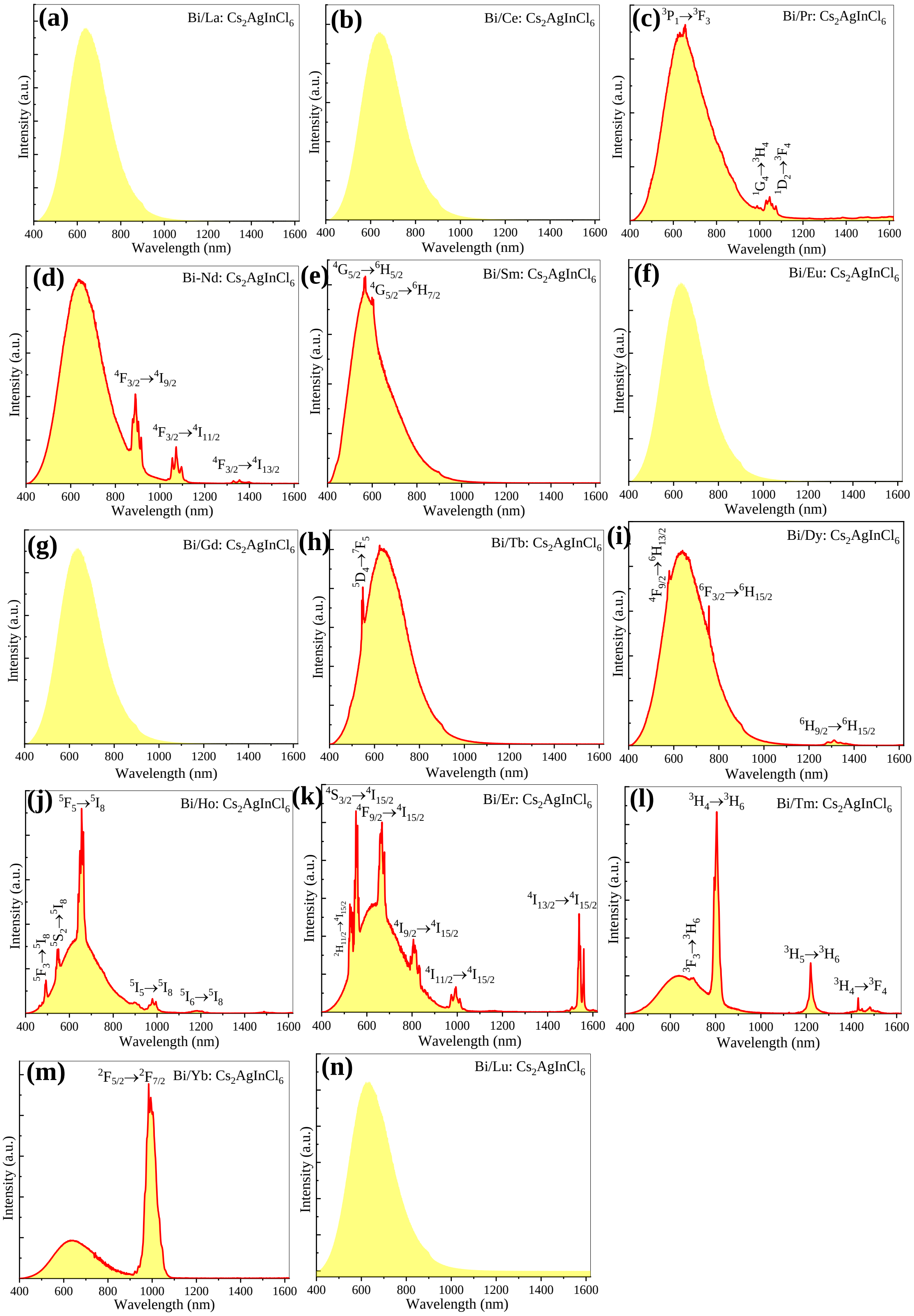

Figure S10. PL spectra recorded from Bi/Ln: $Cs_2AgInCl_6$ samples under the excitation of 350 nm UV light: a) La, b) Ce, c) Pr, d) Nd, e) Sm, f) Eu, g) Gd, h) Tb, i) Dy, j) Ho, k) Er, l) Tm, m) Yb and n) Lu.

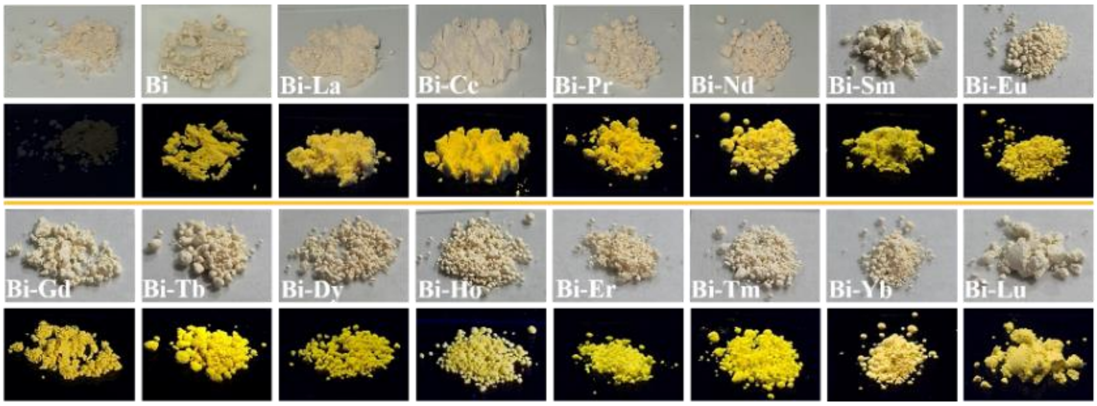

Figure S11. Photographs of Bi/Ln: DPs under daylight and UV light excitation.

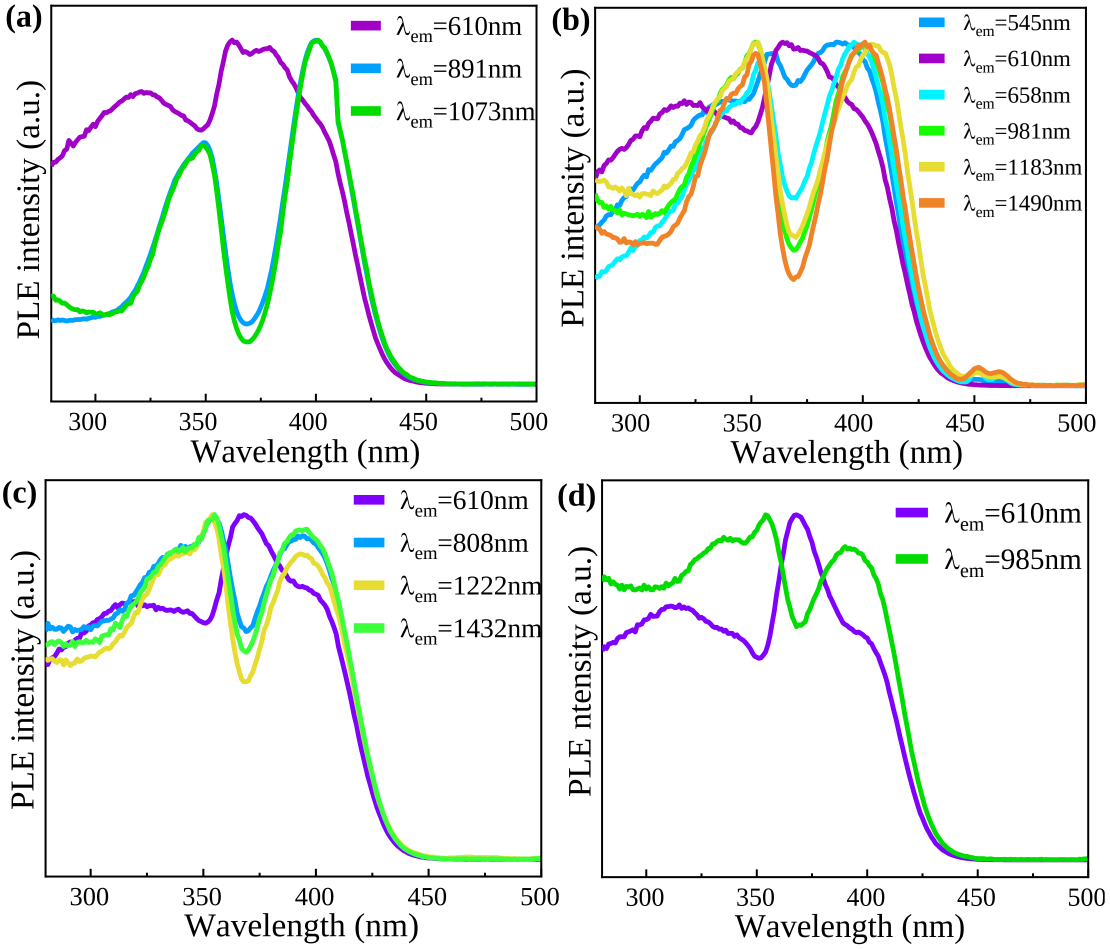

Figure S12. PLE spectra of Bi/Ln: $Cs_2AgInCl_6$ samples by monitoring different emitting wavelengths: a) Ln=Nd, b) Ln=Ho, c) Ln=Tm and d) Ln=Yb.

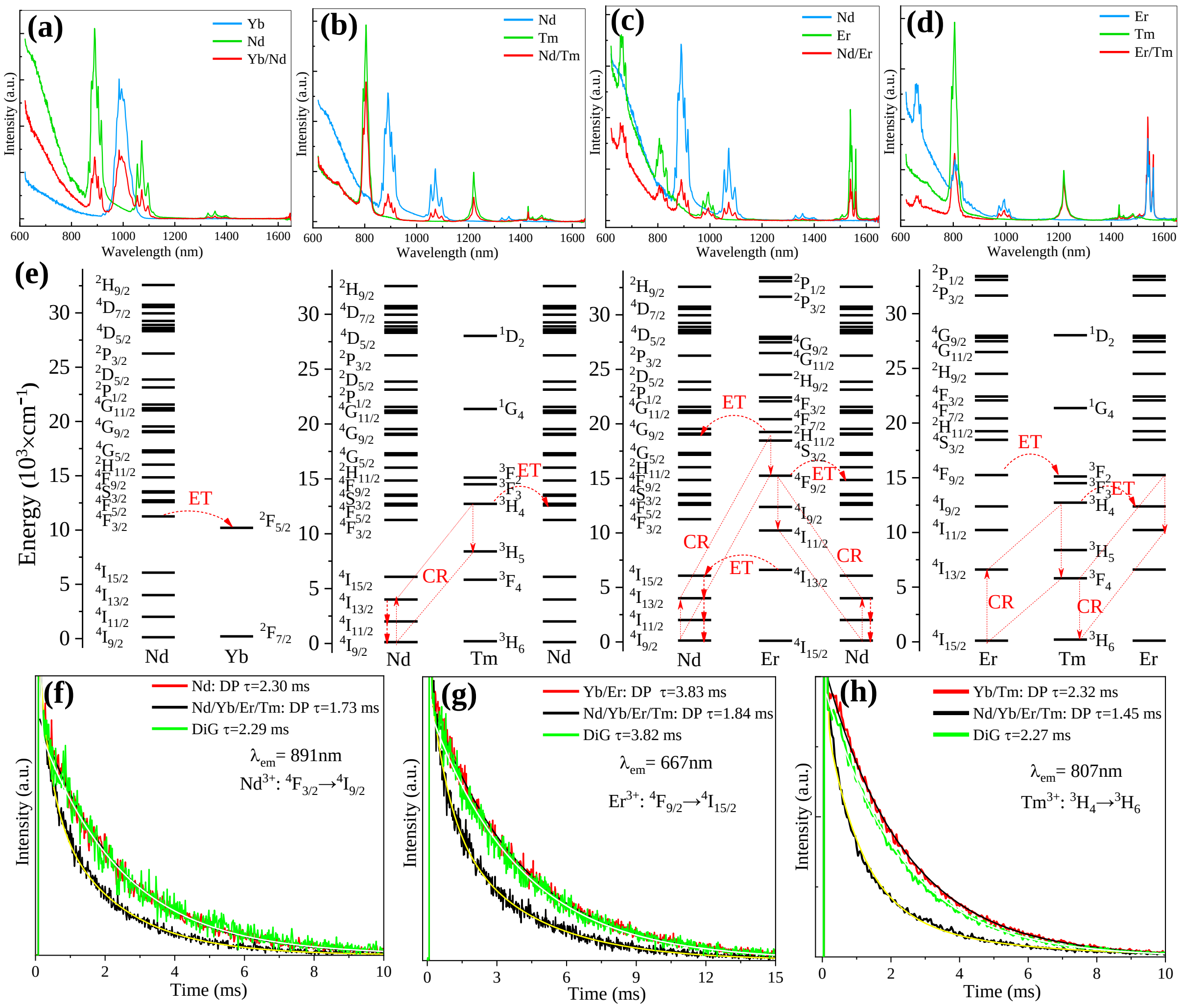

Figure S13. NIR PL spectra recorded from Ln^{3+} single doped and co-doped samples: a) Yb/Nd, b) Nd/Tm, c) Nd/Er and d) Er/Tm, showing the occurrence of PL co-quenching owing to detrimental energy transfers and migrations among Nd^{3+}, Yb^{3+}, Er^{3+} and Tm^{3+} dopants for their well-matching abundant multiplets. e) The proposed several possible energy transfer (ET) and cross relaxation (CR) processes to be responsible for the PL quenching of Ln^{3+} activators. PL decay curves and the fitted lifetimes for the Nd-doped, Yb/Er-doped, Yb/Tm-doped, Nd/Yb/Er/Tm-doped DPs and DiG composite by co-dispersing Nd: DP, Yb/Er: DP and Yb/Tm DP in the same glass matrix by monitoring (f) Nd^{3+}: $^4F_{3/2}$→$^4I_{9/2}$, (g) Er^{3+}: $^4F_{9/2}$→$^4I_{15/2}$ and (h) Tm^{3+}: $^3H_4$→$^3H_6$ transitions.

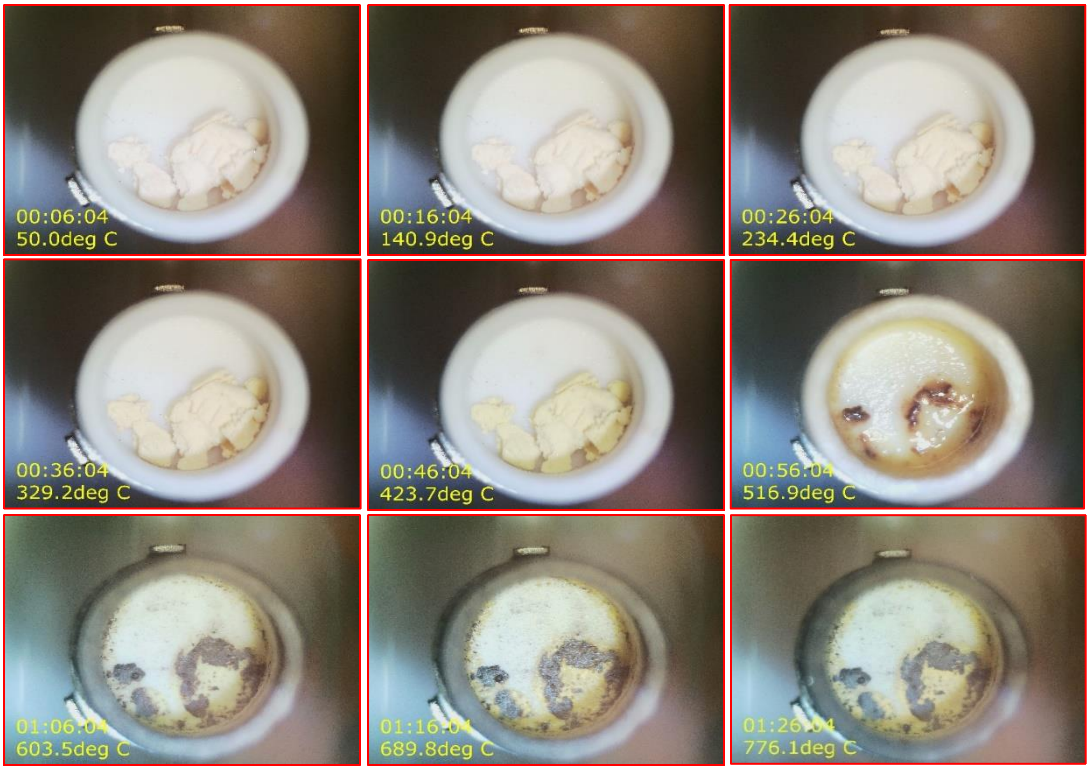

Figure S14. The change in the appearance of a typical Bi/Er: $Cs_2AgInCl_6$ sample with elevation of heating temperature from 50 °C to 776 °C.

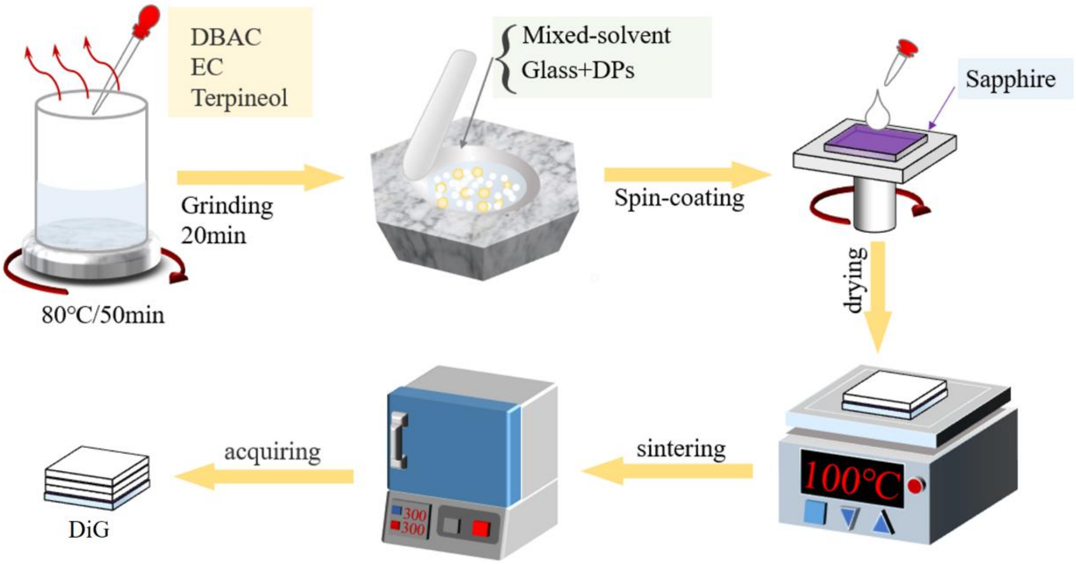

Figure S15. A schematic procedure for preparing multiple Ln: $Cs_2AgInCl_6$ DPs in inorganic glass (DiG).

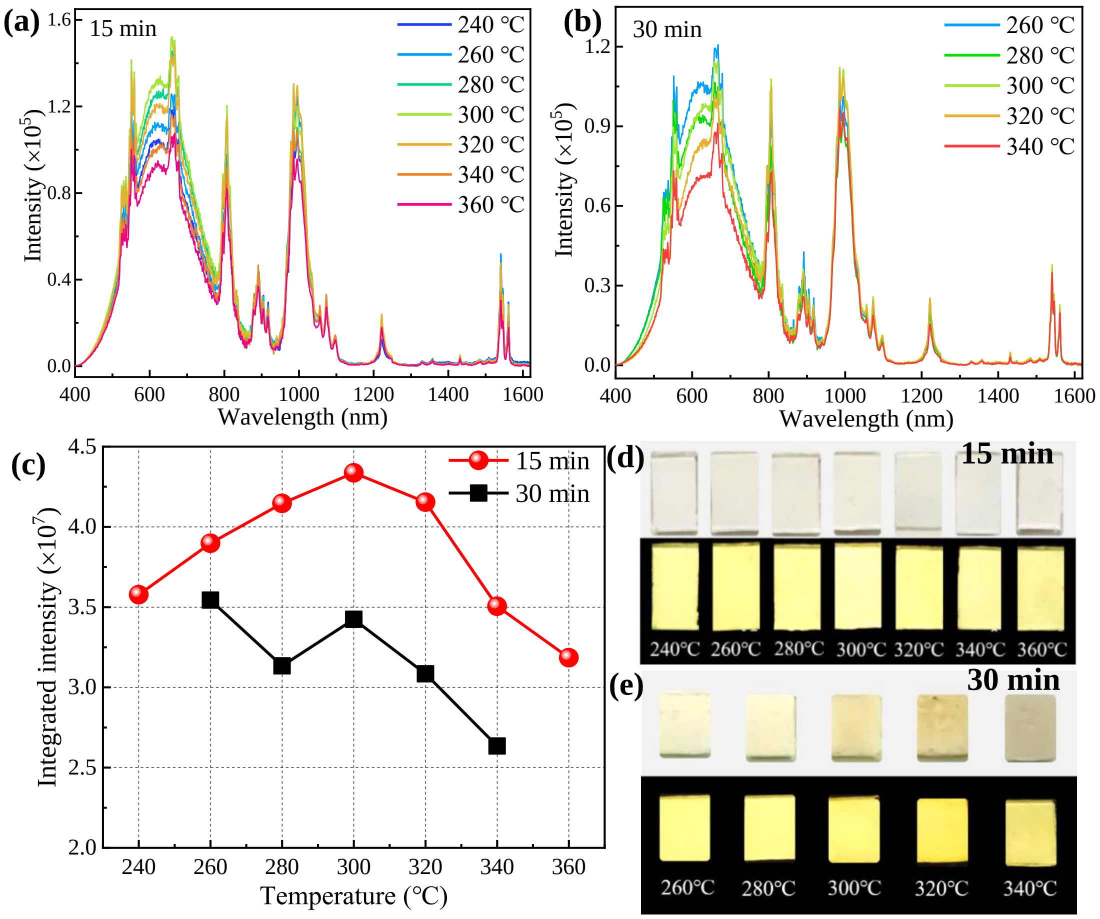

Figure S16. Dependence of PL spectra of the DiG-SA composites on the reaction temperature for a) 15 min and b) 30 min. c) The variation of integrated PL intensity versus reaction temperature. d, e) Photographs of the DiG-SA composites under daylight and UV light excitation.

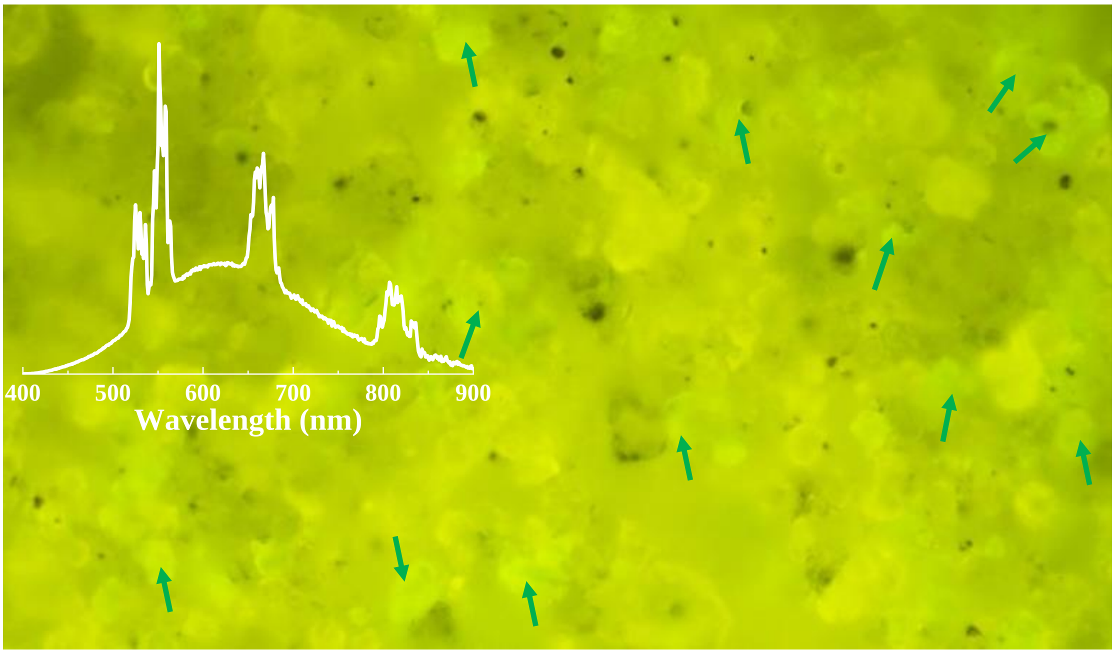

Figure S17. Fluorescence image of Bi/Nd: DP, Bi/Yb/Er: DP and Bi/Yb/Tm: DP co-embedded glass (DiG composite film) under UV light excitation. The Bi/Yb/Er: $Cs_2AgInCl_6$ microcrystals can be distinguished from Bi/Nd: DP and Bi/Yb/Er: DP ones for their light green luminescence (indicated by the arrows) owing to the superimposed Er^{3+} emissions on the STE broadband emission (as shown in the inset PL spectrum).

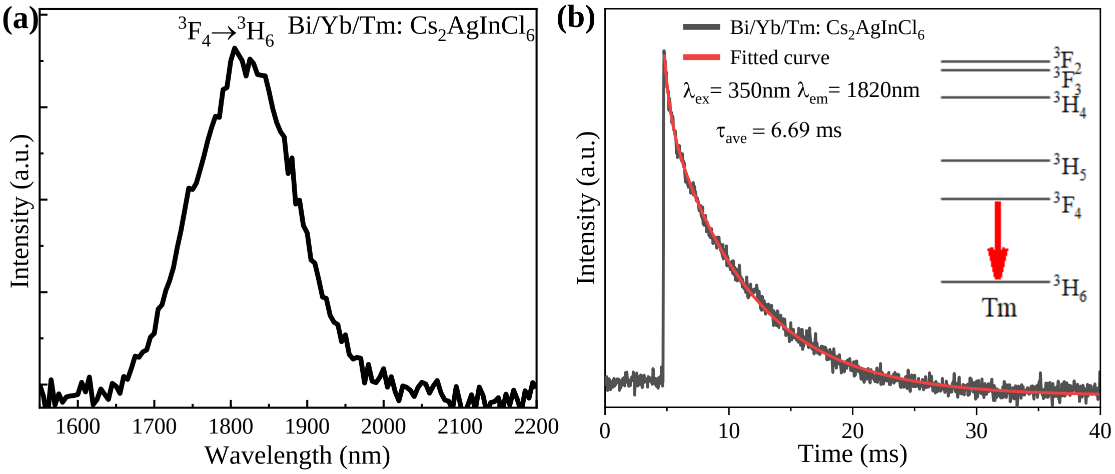

Figure S18. a) NIR PL spectrum and b) decay curve of of Bi/Yb/Tm: $Cs_2AgInCl_6$ sample with the fitted lifetime of 6.69 ms. Inset is energy level diagram showing Tm^{3+}: $^3F_4 \rightarrow ^3H_6$ transition.

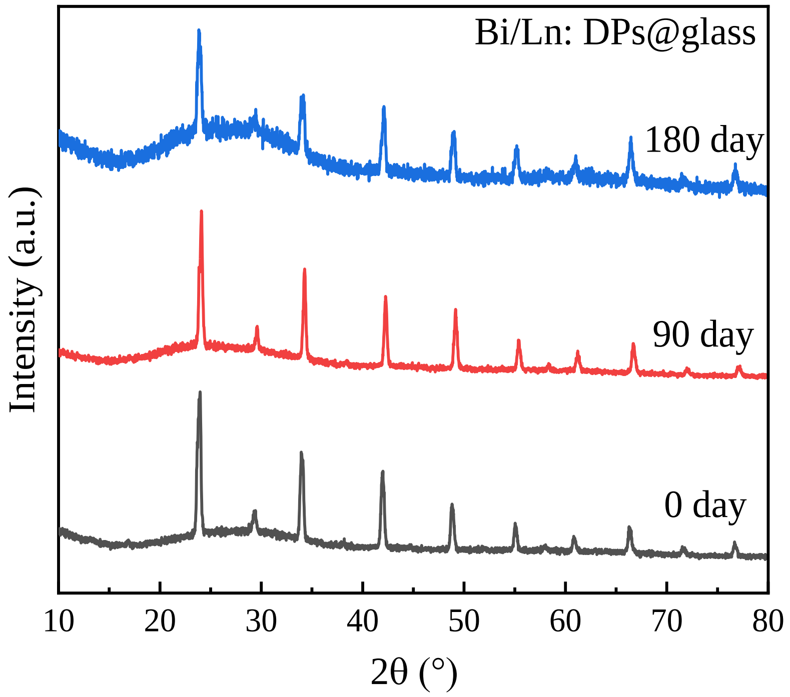

Figure S19. XRD patterns of the fresh DiG sample and the composite after exposing in air for 90 days and 180 days.

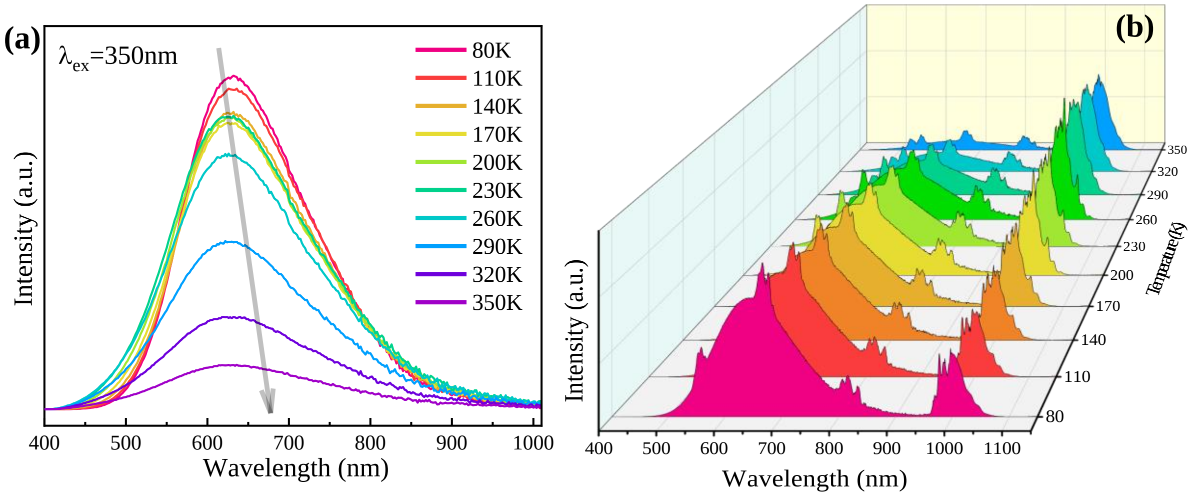

Figure S20. Temperature (80~350 K) dependent PL spectra of (a) the Bi: $Cs_2AgInCl_6$ and (b) Bi/Yb/Er: $Cs_2AgInCl_6$ samples.

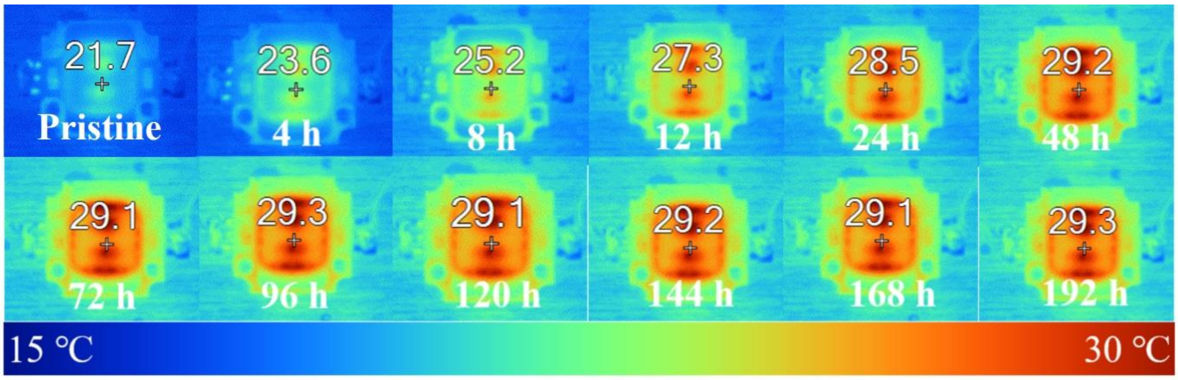

Figure S21. Infrared thermal images recorded from the UV chip after long-term operation (0~192 h).

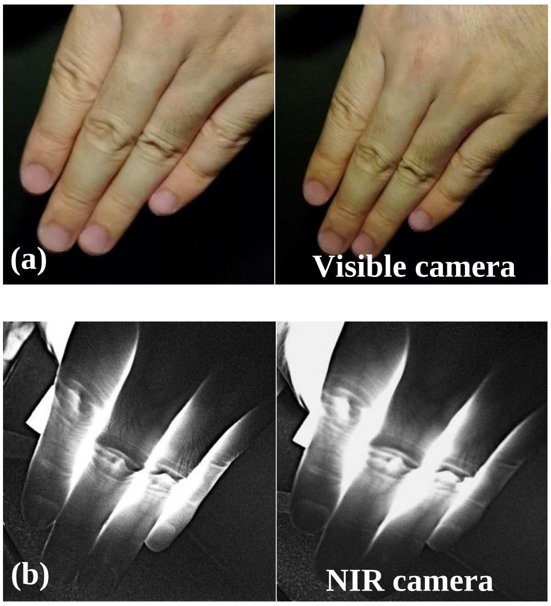

Figure S22. Photographs of palms a) with and b) without u-LED light recorded by the visible camera and NIR camera.

References

[1] M. M. Yao, L. Wang, J. S. Yao, K. H. Wang, C. Chen, B. S. Zhu, J. N. Yang, J. J. Wang, W. P. Xu, Q. Zhang, H. B. Yao, Improving lead-free double perovskite $Cs_2NaBiCl_6$ nanocrystal optical properties via ion doping, Adv. Opt. Mater. 8 (2020) 1901919.

[2] F. Schmitz, K. P. Guo, J. Horn, R. Sorrentino, G. Conforto, F. Lamberti, R. Brescia, F. Drago, M. Prato, Z. B. He, U. Giovanella, F. Cacialli, D. Schlettwein, D. Meggiolaro, T. Gatti, Lanthanide-induced photoluminescence in lead-free $Cs_2AgBiBr_6$ bulk perovskite: insights from optical and theoretical investigations, J. Phys. Chem. Lett. 11 (2020) 8893-8900.

[3] N. Chen, T. Cai, W. Li, K. Hills-Kimball, H. Yang, M. Que, Y. Nagaoka, Z. Liu, D. Yang, A. Dong, C. Y. Xu, R. Zia, Yb- and Mn-doped lead-free double perovskite Cs_2AgBiX_6 (X = Cl^-, Br^-) nanocrystals, ACS Appl. Mater. Interfaces 11 (2019) 16855-16863.

[4] Z. Wang, X. Xu, S. Wang, H. Xu, W. Xu, Q. Zeng, G. Deng, Y. Jiang, S. F. Wu, Cerium doping double perovskite scintillator for sensitive X-ray detection and imaging, Chem. Eur. J. 27 (2021) 9071-9076.

[5] R. Zhang, Z. Wang, X. Xu, X. Mao, J. Xiong, Y. Yang, K. L. Han, All-inorganic rare-Earth halide double perovskite single crystals with highly efficient photoluminescence, Adv. Opt. Mater. 2021 DOI: 10.1002/adom.202100689.

[6] Z. Zeng, B. Huang, X. Wang, L. Lu, Q. Lu, M. Sun, T. Wu, T. Ma, J. Xu, Y. Xu, S. Wang, Y. Du, C. Yan, Multimodal luminescent $Yb^{3+}/Er^{3+}/Bi^{3+}$-doped perovskite single crystals for X-ray detection and anti-counterfeiting, Adv. Mater. 32 (2020) 2004506.

[7] G. Zhang, Y. Wei, P. Dang, H. Xiao, D. Liu, X. Li, Z. Cheng, J. Lin, Facile solution synthesis of Bi^{3+}/Yb^{3+} ions co-doped $Cs_2Na_{0.6}Ag_{0.4}InCl_6$ double perovskites with near-infrared emission, Dalton Trans. 49 (2020) 15231-15237.

[8] C. Wang, P. Liang, R. Xie, Y. Yao, P. Liu, Y. Yang, J. Hu, L. Shao, X. Sun, F. Kang, G. Wei, Highly efficient lead-free (Bi,Ce)-codoped $Cs_2Ag_{0.4}Na_{0.6}InCl_6$ double perovskites for white light-emitting diodes, Chem. Mater. 32 (2020) 7814-7821.

[9] S. Yang, S. Gong, Z. Zhou, L. Wu, M. Zhang, L. Jiang, W. Wu, Bi and Yb codoped $Cs_2Ag_{0.6}Na_{0.4}InCl_6$ microcrystals: visible to near-infrared fluorescence for thermometry, J. Phys. Chem. C. 125 (2021) 10431-10440.

[10] S. Li, Q. Hu, J. Luo, T. Jin, J. Liu, J. Li, Z. Tan, Y. Han, Z. Zheng, T. Zhai, H. Song, L. Gao, G. Niu, J. Tang, Self-trapped exciton to dopant energy transfer in rare earth doped lead-free double perovskite, Adv. Opt. Mater. 7 (2019) 1901098.

[11] W. Lee, S. Hong, S. Kim, Colloidal synthesis of lead-free silver-indium double-perovskite $Cs_2AgInCl_6$ nanocrystals and their doping with lanthanide ions, J. Phys. Chem. C. 123 (2019) 2665-2672.

[12] H. Yin, Q. Kong, R. Zhang, D. Zheng, B. Yang, K. L. Han, Lead-free rare-earth double perovskite $Cs_2AgIn_{1-\gamma-x}Bi_xLa_\gamma Cl_6$ nanocrystals with highly efficient warm-white emission, Sci. China Mater. 64 (2021) 2667-2674.

[13] Y. Liu, X. Rong, M. Li, M. S. Molokeev, J. Zhao, Z. G. Xia, Incorporating rare-earth terbium(III) ions into $Cs_2AgInCl_6$:Bi nanocrystals toward tunable photoluminescence, Angew. Chem. Int. Ed. 59 (2020) 11634-11640.

[14] H. Arfin, J. Kaur, T. Sheikh, S. Chakraborty, A. Nag, Bi^{3+}-Ln^{3+} (Ln = Er and Yb) codoped $Cs_2AgInCl_6$ double perovskite near infrared emitter, Angew. Chem. Int. Ed. 59 (2020) 11307-11311.

[15] Y. Mahor, W. J. Mir, A. Nag, Synthesis and near-infrared emission of Yb-doped $Cs_2AgInCl_6$ double perovskite microcrystals and nanocrystals, J. Phys. Chem. C. 123 (2019) 15787-15793.
